# Axiomatic Information Thermodynamics

**DOI:** 10.3390/e20040237

**Published:** 2018-03-29

**Authors:** Austin Hulse, Benjamin Schumacher, Michael D. Westmoreland

**Affiliations:** 1 Department of Physics, Kenyon College, Gambier, OH 43022, USA; 2 Department of Mathematics, Denison University, Granville, OH 43023, USA

**Keywords:** axiomatic thermodynamics, information, entropy, Maxwell’s demon

## Abstract

We present an axiomatic framework for thermodynamics that incorporates information as a fundamental concept. The axioms describe both ordinary thermodynamic processes and those in which information is acquired, used and erased, as in the operation of Maxwell’s demon. This system, similar to previous axiomatic systems for thermodynamics, supports the construction of conserved quantities and an entropy function governing state changes. Here, however, the entropy exhibits both information and thermodynamic aspects. Although our axioms are not based upon probabilistic concepts, a natural and highly useful concept of probability emerges from the entropy function itself. Our abstract system has many models, including both classical and quantum examples.

## 1. Introduction

Axiomatic approaches to physics are useful for exploring the conceptual basis and logical structure of a theory. One classic example was presented by Robin Giles over fifty years ago in his monograph *Mathematical Foundations of Thermodynamics* [1]. His theory is constructed upon three phenomenological concepts: thermodynamic states, an operation (+) that combines states into composites, and a relation (→) describing possible state transformations. From a small number of basic axioms, Giles derives a remarkable amount of thermodynamic machinery, including conserved quantities (“components of content”), the existence of an entropy function that characterizes irreversibility for possible processes, and so on.

Alternative axiomatic developments of thermodynamics have been constructed by others along different lines. One notable example is the framework of Lieb and Yngvason [2,3] (which has recently been used by Thess as the basis for a textbook [4]). Giles’s abstract system, meanwhile, has found application beyond the realm of classical thermodynamics, e.g., in the theory of quantum entanglement [5].

Other work on the foundations of thermodynamics has focused on the concept of information. Much of this has been inspired by Maxwell’s famous thought-experiment of the demon [6]. The demon is an entity that can acquire and use information about the microscopic state of a thermodynamic system, producing apparent violations of the Second Law of Thermodynamics. These violations are only “apparent” because the demon is itself a physical system, and its information processes are also governed by the underlying dynamical laws.

**Figure 1:**
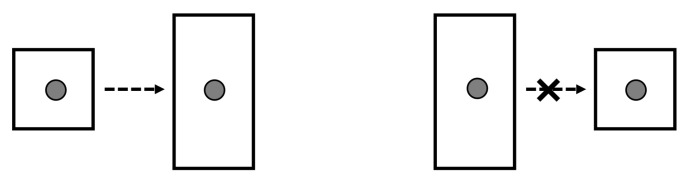
A one-particle gas may freely expand to occupy a larger volume, but the reverse process would violate the Second Law.

Let us examine a highly simplified example of the demon at work. Our thermodynamic system is a one-particle gas enclosed in a container (a simple system also used in [7]). The gas may be allowed to freely expand into a larger volume, but this process is irreversible. A “free compression” process that took the gas from a larger to a smaller volume with no other net change would decrease the entropy of the system and contradict the Second Law. See Figure 1.

**Figure 2:**
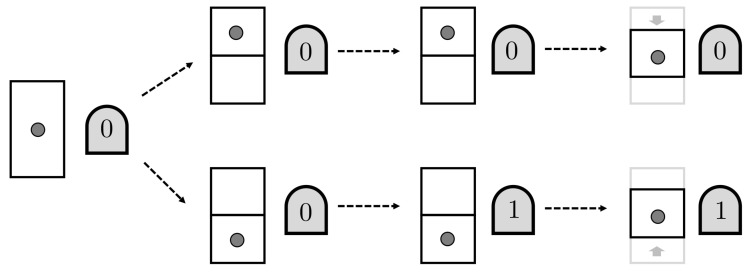
A Maxwell’s demon device acquires one bit of information about the location of a gas particle, allowing it to contract the gas to a smaller volume.

Now, we introduce the demon, which is a machine that can interact with the gas particle and acquire information about its location. The demon contains a one-bit memory register, initially in the state 0. First, a partition is introduced in the container, so that the gas particle is confined to the upper or lower half (*U* or *L*). The demon measures the gas particle and records its location in memory, with 0 standing for *U* and 1 for *L*. On the basis of this memory bit value, the demon moves the volume containing the particle. In the end, the gas particle is confined to one particular smaller volume, apparently reducing the entropy of the system. This is illustrated in Figure 2.

As Bennett pointed out [8], every demon operation we have described can be carried out reversibly, with no increase in global entropy. Even the “measurement” process can be described by a reversible interaction between the particle location and the demon’s memory bit *b*, changing the states according to

(1) $$\left(U,b\right)⤎⤏\left(U,b\right)\;\left(L,b\right)⤎⤏\left(L,\bar{b}\right),$$

where $\bar{b}$ is the binary negation of *b*. However, the demon process as described leaves the demon in a new situation, since an initially blank memory register now stores a bit of information. To operate in a cycle (and thus unambiguously violate the Second Law), the demon must erase this bit and recover the blank memory. However, as Landauer showed [9], the erasure of a bit of information is always accompanied by an entropy increase of at least $k_{B}\ln2$ in the surroundings, just enough to ensure that there is no overall entropy decrease in the demon’s operation. The Second Law remains valid.

Information erasure is a physical process. The demon we have described can also erase a stored bit simply by reversing the steps outlined. Depending on the value of the bit register, the demon moves the gas particle to one of two corresponding regions separated by a partition. The same interaction that accomplished the “measurement” process (which takes (U,0)⤏(U,0) and (L,0)⤏(L,1)) can now be used to reset the bit value to 0 (taking (U,0)⤏(U,0) and (L,1)⤏(L,0)). In other words, the information stored redundantly in both the gas and the register is “uncopied”, so that it remains only in the gas. Finally, the partition is removed and the gas expands to the larger volume. The net effect is to erase the memory register while increasing the entropy of the gas by an amount $k_{B}\ln2$, in conventional units.

Another link between thermodynamics and information comes from statistical mechanics. As Jaynes has shown [10,11], the concepts and measures of information devised by Shannon for communication theory [12] can be used in the statistical derivation of macroscopic thermodynamic properties. In a macroscopic system, we typically possess only a small amount of information about a few large-scale parameters (total energy, volume, etc.). According to Jaynes, we should therefore choose a probability distribution over microstates that maximizes the Shannon entropy, consistent with our data. That is to say, the rational probability assignment includes no information (in the Shannon sense) except that found in the macroscopic state of the system. This prescription yields the usual distributions used in statistical mechanics, from which thermodynamic properties may be derived.

Axiomatic theories and information analyses each provide important insights into the meaning of thermodynamics. The purpose of this paper is to synthesize these two approaches. We will present an axiomatic basis for thermodynamics that uses information ideas from the very outset. In such a theory, Maxwell’s demon, which accomplishes state changes by acquiring information, is neither a paradox nor a sideshow curiosity. Instead, it is a central conceptual tool for understanding the transformations between thermodynamic states. In our view, thermodynamics is essentially a theory of the descriptions of systems possessed by agents that are themselves (like the demon) physical systems. These descriptions may change as the external systems undergo various processes; however, they also may change when the agent acquires, uses or discards information.

Our work is thus similar in spirit to that of Weilenmann et al. [13], though our approach is very different. They essentially take the Lieb-Yngvason axiomatic framework and apply it to various quantum resource theories. In particular, for the resource theory of non-uniformity [14], the Lieb-Yngvason entropy function coincides with the von Neumann entropy of the quantum density operator, which is a measure of quantum information. We, on the other hand, seek to modify axiomatic thermodynamics itself to describe processes involving “information engines” such as Maxwell’s demon. We do not rely on any particular microphysics and have both classical and quantum models for our axioms. The connections we find between information and thermodynamic entropy are thus as general as the axioms themselves. Furthermore, to make our concept of information as clear as possible, we will seek to base our development on the most elementary ideas of state and process.

We therefore take as our prototype the axiomatic theory of Giles [1]. In fact, Giles’s monograph contains two different axiomatic developments. (The first system is presented in Chapters 1–6 of Giles’s book, and the second is introduced beginning in Chapter 7.) The first, which we might designate Giles I, is based on straightforward postulates about the properties of states and processes. The second, Giles II, is more sophisticated and powerful. The axioms are less transparent in meaning (e.g., the assumed existence of “anti-equilibrium” and “internal” thermodynamic states), but they support stronger theorems. This difference can be illustrated by the status of entropy functions in the two developments. In Giles I, it is shown that an entropy function exists; in fact, there may be many such functions. In Giles II, it is shown that an *absolute* entropy function, one that is zero for every anti-equilibrium state, exists and is unique. Giles himself regarded the second system as “The Formal Theory”, which he summarizes in an Appendix of that title.

The information-based system we present here, on the other hand, is closer to the more elementary framework of Giles I. We have taken some care to use notation and concepts that are as analogous as possible to that theory. We derive many of Giles’s propositions, including some that he uses as axioms. Despite the similarities, however, even the most elementary ideas (such as the combination of states represented by the + operation) will require some modifications. These changes highlight the new ideas embodied in axiomatic information thermodynamics, and so we will take some care to discuss them as they arise.

Section 2 introduces the fundamental idea of an eidostate, including how two or more eidostates may be combined. In Section 3, we introduce the → relation between eidostates: A→B means that eidostate *A* can be transformed into eidostate *B*, with no other net change in the apparatus that accomplishes the transformation. The collection of processes has an algebraic structure, which we present in Section 4. We introduce our concept of information in Section 5 and show that the structure of information processes imposes a unique entropy measure on pure information states.

Section 6 introduces axioms inspired by Maxwell’s demon and shows their implications for processes involving thermodynamic states. Section 7 and Section 8 outline how our information axioms also yield Giles’s central results about thermodynamic processes, including the existence of an entropy function, conserved components of content, and mechanical states. In Section 10, we see that an entropy function can be extended in a unique way to a wider class of “uniform” eidostates. This, rather remarkably, gives rise to a unique probability measure within uniform eidostates, as we describe in Section 11.

Section 12 and Section 13 present two detailed models of our axioms, each one highlighting a different aspect of the theory. We conclude in Section 14 with some remarks on the connection between information and thermodynamic entropy, a connection that emerges necessarily from the structure of processes in our theory.

## 2. Eidostates

Giles [1] bases his development on a set S of *states*. The term “state” is undefined in the formal theory, but heuristically it represents an equilibrium macrostate of some thermodynamic system. (Giles, in fact, identifies a∈S with some method of preparation; the concept of a “system” does not appear in his theory, or in ours.) States can be combined using the + operation, so that if a,b∈S then a+b∈S also. The new state a+b is understood as the state of affairs arising from simultaneous and independent preparations of states *a* and *b*. For Giles, this operation is commutative and associative; for example, a+b is exactly the same state as b+a.

We also have a set S. However, our theory differs in two major respects. First, the combination of states is not assumed to be commutative and associative. On the contrary, we regard a+b and b+a as entirely distinct states for any a≠b. The motivation for this is that the way that a composite state is assembled encodes information about its preparation, and we want to be able to keep track of this information. On the states in S, the operation + is simply that of forming a Cartesian pair of states: a+b=(a,b), and nothing more.

A second and more far-reaching difference is that we cannot confine our theory to individual elements of S. A theory that keeps track of information in a physical system must admit non-deterministic processes. For instance, if a measurement is made, the single state *a* before the measurement may result in any one of a collection of possible states $\left\{a_{0},a_{1},\ldots ,a_{n}\right\}$ corresponding to the various results.

Therefore, our basic notion is the *eidostate*, which is a finite nonempty set of states. The term “eidostate” derives from the Greek word *eidos*, meaning “to see”. The eidostate is a collection of states that may be regarded as possible from the point of view of some agent. The set of eidostates is designated by E. When we combine two eidostates A,B∈E into the composite A+B, we mean the set of all combinations a+b of elements of these sets. That is, A+B is exactly the Cartesian product (commonly denoted A×B) for the sets. Thus, our state combination operation + is very different from that envisioned by Giles. His operation is an additional structure on S that requires an axiom to fix its properties, whereas we simply use the Cartesian product provided by standard set theory, which we of course assume.

Some eidostates in E are Cartesian products of other sets (which are also eidostates); some eidostates are not, and are thus “prime”. We assume that each eidostate has a “prime factorization” into a finite number of components. Here, is our first axiom:

**Axiom** **1.** *Eidostates: E is a collection of sets called* eidostates *such that:*

*(a)*  Every A∈E is a finite nonempty set with a finite prime Cartesian factorization.
*(b)*  A+B∈E *if and only if A,B∈E.*
*(c)*  Every nonempty subset of an eidostate is also an eidostate.

Part (c) of this axiom ensures, among other things, that every element *a* of an eidostate can be associated with a singleton eidostate {a}. Without too much confusion, we can simply denote any singleton eidostate {a} by the element it contains, writing *a* instead. We can therefore regard the set of states S in two ways. Either we may think of S as the collection of singleton eidostates in E, or we may say that S is the collection of all elements of all eidostates: $S=\underset{A\in E}{⋃}A$. Either way, the set E characterized by Axiom 1 is the more fundamental object.

Our “eidostates” are very similar to the “specifications” introduced by del Rio et al. [15] in their general framework for resource theories of knowledge. The two ideas, however, are not quite identical. To begin with, a specification *V* may be any subset of a state space Ω, whereas the eidostates in E are required to be finite nonempty subsets of S—and indeed, not every such subset need be an eidostate. For example, the union A∪B of two eidostates is not necessarily an eidostate. The set E therefore does not form a Boolean lattice. Specifications are a general concept applicable to state spaces of many different kinds, and are used in [15] to analyze different resource theories and to express general notions of approximation and locality. We, however, will be restricting our attention to an E (and S) with very particular properties, expressed by Axiom 1 and our later axioms, that are designed to model thermodynamics in the presence of information engines such as Maxwell’s demon.

Composite eidostates are formed by combining eidostates with the + operation (Cartesian product). The same pieces may be combined in different ways. We say that A,B∈E are *similar* (written A∼B) if they are made up of the same components. That is, A∼B provided there are eidostates $E_{1},\ldots ,E_{n}$ such that $A=F_{A}\left(E_{1},\ldots ,E_{n}\right)$ and $B=F_{B}\left(E_{1},\ldots ,E_{n}\right)$ for two Cartesian product formulas $F_{A}$ and $F_{B}$. Thus,

(2) $$\left(E_{1}+E_{2}\right)+E_{3}∼E_{2}+\left(E_{1}+E_{3}\right),$$

and so on. The similarity relation ∼ is an equivalence relation on E.

We sometimes wish to combine an eidostate with itself several times. For the integer n≥1, we denote by nA the eidostate A+(A+…), where *A* appears *n* times in the nested Cartesian product. This is one particular way to combine the *n* instances of *A*, though of course all such ways are similar. Thus, we may assert equality in A+nA=(n+1)A, but only similarity in nA+A∼(n+1)A.

Finally, we note that we have introduced as yet no probabilistic ideas. An eidostate is a simple enumeration of possible states, without any indication that some are more or less likely than others. However, as we will see in Section 11, in certain contexts, a natural probability measure for states does emerge from our axioms.

## 3. Processes

In the axiomatic thermodynamics of Giles, the → relation describes state transformations. The relation a→b means that there exists another state *z* and a definite time interval τ≥0 so that

(3) $$a+z\overset{\tau }{⇝}b+z,$$

where $\overset{\tau }{⇝}$ indicates time evolution over the period τ. The pair (z,τ) is the “apparatus” that accomplishes the transformation from *a* to *b*. This dynamical evolution is a deterministic process; that is, in the presence of the apparatus (z,τ) the initial state *a* is guaranteed to evolve to the final state *b*. This rule of interpretation for → motivates the properties assumed for the relation in the axioms.

Our version of the arrow relation → is slightly different, in that it encompasses non-deterministic processes. Again, we envision an apparatus (z,τ) and we write

(4) $$a+z\overset{\tau }{⤏}b+z,$$

to mean that the initial state a+z may possibly evolve to b+z over the stated interval. Then, for eidostates *A* and *B*, the relation A→B means that, if a∈A and b∈S, then there exists an apparatus (z,τ) such that $a+z\overset{\tau }{⤏}b+z$ only if b∈B. Each possible initial state *a* in *A* might evolve to one or more final states, but all of the possible final states are contained in *B*. For singleton eidostates *a* and *b*, the relation a→b represents a deterministic process, as in Giles’s theory.

Again, we use our heuristic interpretation of → to motivate the essential properties specified in an axiom:

**Axiom** **2.** *Processes: Let eidostates A,B,C∈E, and s∈S.*

*(a)*  If A∼B, then A→B.
*(b)*  If A→B and B→C, then A→C.
*(c)*  If A→B, then A+C→B+C.
*(d)*  If A+s→B+s, then A→B.

Part (a) of the axiom asserts that it is always possible to “rearrange the pieces” of a composite state. Thus, A+B→B+A and so on. Of course, since ∼ is a symmetric relation, A∼B implies both A→B and B→A, which we might write as A↔B.

Part (b) says that a process from *A* to *C* may proceed via an intermediate eidostate *B*. Parts (c) and (d) establish the relationship between → and +. We can always append a “bystander” state *C* to any process A→B, and a bystander singleton state *s* in A+s→B+s can be viewed as part of the apparatus that accomplishes the transformation A→B.

We use the → relation to characterize various conceivable processes. A *formal process* is simply a pair of eidostates $\langle A,B\rangle$. Following Giles, we may say that $\langle A,B\rangle$ is:

- *natural* if A→B;
- *antinatural* if B→A;
- *possible* if A→B or B→A (which may be written A⇌B);
- *impossible* if it is not possible;
- *reversible* if A↔B; and
- *irreversible* if it is possible but not reversible.

Thus, any formal process must be one of four *types*: reversible, natural irreversible, antinatural irreversible, or impossible.

Any nonempty subset of an eidostate is an eidostate. If the eidostate *A* is an enumeration of possible states, the proper subset eidostate B⊊A may be regarded as an enumeration with some additional condition present that eliminates one or more of the possibilities. What can we say about processes involving these “conditional” eidostates? To answer this question, we introduce two further axioms. The first is this:

**Axiom** **3.** If A,B∈E and B is a proper subset of A, then A↛B.

This expresses the idea that no natural process can simply eliminate a state from a list of possibilities. This is a deeper principle than it first appears. Indeed, as we will find, in our theory, it is the essential ingredient in the Second Law of Thermodynamics.

To express the second new axiom, we must introduce the notion of a *uniform* eidostate. The eidostate *A* is said to be uniform provided, for every a,b∈A, we have a⇌b. That is, every pair of states in *A* is connected by a possible process. This means that all of the *A* states are “comparable” in some way involving the → relation.

All singleton eidostates are uniform. Are there any non-uniform eidostates? The axioms in fact do not tell us. We will find models of the axioms that contain non-uniform eidostates and others that contain none. Even if there are states a,b∈S for which a↛b and b↛a, nothing in our axioms guarantees the existence of an eidostate that contains both *a* and *b* as elements. We denote the set of uniform eidostates by U⊆E.

Now, we may state the second axiom about conditional processes.

**Axiom** **4.** *Conditional processes:*

*(a)*  Suppose $A,A^{\prime }\in E$ and b∈S. If A→b and $A^{\prime }\subseteq A$ then $A^{\prime }\to b$.
*(b)*  Suppose A and B are uniform eidostates that are each disjoint unions of eidostates: $A=A_{1}\cup A_{2}$ and $B=B_{1}\cup B_{2}$. If $A_{1}\to B_{1}$ and $A_{2}\to B_{2}$ then A→B.

The first part makes sense given our interpretation of the → relation in terms of an apparatus (z,τ). If every a∈A satisfies $a+z\overset{\tau }{⤏}b+z$ for only one state *b*, then the same will be true for every $a\in A^{\prime }\subseteq A$. Part (b) of the axiom posits that, if we can find an apparatus whose dynamics transforms $A_{1}$ states into $B_{1}$ states, and another whose dynamics transforms $A_{2}$ states into $B_{2}$ states, then we can devise a apparatus with “conditional dynamics” that does both tasks, taking *A* to *B*. This is a rather strong proposition, and we limit its scope by restricting it to the special class of uniform eidostates.

We can as a corollary extend Part (b) of Axiom 4 to more than two subsets. That is, suppose A,B∈U and the sets *A* and *B* are each partitioned into *n* mutually disjoint, nonempty subsets: $A=A_{1}\cup \cdots \cup A_{n}$ and $B=B_{1}\cup \cdots B_{n}$. In addition, suppose $A_{k}\to B_{k}$ for all k=1,…,n. Then, A→B.

## 4. Process Algebra and Irreversibility

Following Giles, we now explore the algebraic structure of formal processes and describe how the type of a possible process may be characterized by a single real-valued function. Although the broad outlines of our development will follow that of Giles [1], there are significant differences. (For example, in our theory, the unrestricted set P of eidostate processes does not form a group.)

There is an equivalence relation among formal processes, based on the similarity relation ∼ among eidostates. We say that $\langle A,B\rangle ≐\langle C,D\rangle$ if there exist singletons x,y∈S such that A+x∼C+y and B+x∼D+y. That is, if we augment the eidostates in $\langle A,B\rangle$ by *x* and those in $\langle C,D\rangle$ by *y*, the corresponding eidostates in the two processes are mere rearrangements of each other. It is not hard to establish that this is an equivalence relation. Furthermore, equivalent processes (under ≐) are always of the same type. To see this, suppose that if $\langle A,B\rangle ≐\langle C,D\rangle$ and A→B. Then, A+x∼C+y, etc., and

(5) C+y→A+x→B+x→D+y.

Hence, C→D. It is a straightforward corollary that A→B if and only if C→D.

Processes (or more strictly, equivalence classes of processes under ≐) have an algebraic structure. We define the *sum* of two processes as

(6) $$\langle A,B\rangle +\langle C,D\rangle =\langle A+C,B+D\rangle ,$$

and the *negation* of a process as $-\langle A,B\rangle =\langle B,A\rangle$. We call the − operation “negation” even though in general $-\langle A,B\rangle$ is not the additive inverse of $\langle B,A\rangle$. Such an inverse may not exist. As we will see, however, the negation does yield an additive inverse process in some important special contexts.

The sum and negation operations on processes are both compatible with the equivalence ≐. That is, first, if $\langle A,B\rangle ≐\langle A^{\prime },B^{\prime }\rangle$ then $-\langle A,B\rangle ≐-\langle A^{\prime },B^{\prime }\rangle$. Furthermore, if $\langle A,B\rangle ≐\langle A^{\prime },B^{\prime }\rangle$ and $\langle C,D\rangle ≐\langle C^{\prime },D^{\prime }\rangle$, then

(7) $$\langle A,B\rangle +\langle C,D\rangle ≐\langle A^{\prime },B^{\prime }\rangle +\langle C^{\prime },D^{\prime }\rangle .$$

This means that the sum and negation operations are well-defined on equivalence classes of processes. If $\left[\langle A,B\rangle \right]$ and $\left[\langle C,D\rangle \right]$ denote two equivalence classes represented by $\langle A,B\rangle$ and $\langle C,D\rangle$, then

(8) $$\left[\langle A,B\rangle \right]+\left[\langle C,D\rangle \right]=\left[\langle A+C,B+D\rangle \right],$$

which is the same regardless of the particular representatives chosen to indicate the two classes being added.

We let P denote the collection of all equivalence classes of formal processes. Then, the sum operation both is associative and commutative on P. Furthermore, it contains a zero element $0=\left[\langle s,s\rangle \right]$ for some singleton *s*. That is, if $\Gamma =\left[\langle A,B\rangle \right]$,

(9) $$\Gamma +0=\left[\langle A,B\rangle \right]+\left[\langle s,s\rangle \right]=\left[\langle A+s,B+s\rangle \right]=\left[\langle A,B\rangle \right]=\Gamma .$$

The set P is thus a monoid (a semigroup with identity) under the operation +. Moreover, the subset $P_{S}$ of singleton processes—equivalence classes of formal processes with singleton initial and final states—is actually an Abelian group, since

(10) $$\left[\langle a,b\rangle \right]+\left[\langle b,a\rangle \right]=\left[\langle a+b,b+a\rangle \right]=\left[\langle a+b,a+b\rangle \right]=0.$$

In $P_{S}$, the negation operation does yield the additive inverse of an element.

The → relation on states induces a corresponding relation on processes. If Γ,Δ∈P, we say that Γ→Δ provided the process Γ−Δ is natural (a condition we might formally write as Γ−Δ→0). Intuitively, this means that process Γ can “drive process Δ backward”, so that Γ and the opposite of Δ together form a natural process.

Now, suppose that $\hat{P}$ is a collection of equivalence classes of possible processes that is closed under addition and negation. An *irreversibility function* I is a real-valued function on $\hat{P}$ such that

1. If $\Gamma ,\Delta \in \hat{P}$, then I(Γ+Δ)=I(Γ)+I(Δ).
2. The value of I determines the type of the processes in $\hat{P}$:
  - I(Γ)>0 whenever Γ is natural irreversible.
  - I(Γ)=0 whenever Γ is reversible.
  - I(Γ)<0 whenever Γ is antinatural irreversible.

An irreversibility function, if it exists, has a number of elementary properties. For instance, since the process Γ+(−Γ) is always reversible for any $\Gamma \in \hat{P}$, it follows that I(−Γ)=−I(Γ). In fact, we can show that all irreversibility functions on $\hat{P}$ are essentially the same:

**Theorem** **1.** An irreversibility function on $\hat{P}$ is unique up to an overall positive factor.

**Proof.**  Suppose $I_{1}$ and $I_{2}$ are irreversibility functions on the set $\hat{P}$. If Γ is reversible, then $I_{1}\left(\Gamma \right)=I_{2}\left(\Gamma \right)=0$. If there are no irreversible processes in $\hat{P}$, then $I_{1}=I_{2}$.

Now, suppose that $\hat{P}$ contains at least one irreversible process Γ, which we may suppose is natural irreversible. Thus, both $I_{1}\left(\Gamma \right)>0$ and $I_{2}\left(\Gamma \right)>0$. Consider some other process $\Delta \in \hat{P}$. We must show that

(11) $$\frac{I_{1}\left(\Delta \right)}{I_{1}\left(\Gamma \right)}=\frac{I_{2}\left(\Delta \right)}{I_{2}\left(\Gamma \right)}.$$

We proceed by contradiction, imagining that the two ratios are not equal and (without loss of generality) that the second one is larger. Then, there exists a rational number m/n (with n>0) such that

(12) $$\frac{I_{1}\left(\Delta \right)}{I_{1}\left(\Gamma \right)}<\frac{m}{n}<\frac{I_{2}\left(\Delta \right)}{I_{2}\left(\Gamma \right)}.$$

The first inequality yields $mI_{1}\left(\Gamma \right)-nI_{1}\left(\Delta \right)>0$, so that the process mΓ−nΔ (that is, mΓ+n(−Δ)) must be natural irreversible. The second inequality yields $mI_{2}\left(\Gamma \right)-nI_{2}\left(\Delta \right)<0$, so that the process mΓ−nΔ must be antinatural irreversible. These cannot both be true, so the original ratios must be equal. ☐

The additive irreversibility function I is only defined on a set $\hat{P}$ of possible processes. However, we can under some circumstances extend an additive function to a wider domain. It is convenient to state here the general mathematical result we will use later for this purpose:

**Theorem** **2** (“Hahn–Banach theorem” for Abelian groups)**.** Let G be an Abelian group. Let ϕ be a real-valued function defined and additive on a subgroup $G_{0}$ of G. Then, there exists an additive function $\phi^{\prime }$ defined on G such that $\phi^{\prime }\left(x\right)=\phi \left(x\right)$ for all x in $G_{0}$.

Note that the extension $\phi^{\prime }$ is not necessarily unique—that is, there may be many different extensions of a single additive function ϕ.

## 5. Information and Entropy

In our theory, *information* resides in the distinction among possible states. Thus, the eidostate $A=\{a_{1},a_{2},a_{3}\}$ represents information in the distinction among it elements. However, thermodynamic states such as the $a_{k}$s may also have other properties such as energy, particle content, and so on. To disentangle the concept of information from the other properties of these states, we introduce a notion of a “pure” information state.

The intuitive idea is this. We imagine that the world contains freely available memory devices. Different configurations of these memories—different memory records—are distinct states that are degenerate in energy and every other conserved quantity. Any particular memory record can thus be freely created from or reset to some null value.

We therefore define a *record state* to be an element r∈S such that there exists a∈S so that a↔a+r. The state *a* can be thought of as part of the apparatus that reversibly exchanges the particular record *r* with a null value. In fact, if *A* is any eidostate at all, we find that

(13) A+a↔A+(a+r)↔(A+r)+a,

and so by cancellation of the singleton state *a*, A↔A+r. We denote the set of record states by R. If r,s∈R, then r+s∈R, and furthermore r↔s. Any particular record state can be reversibly transformed into any other, a fact that expresses the arbitrariness of the “code” used to represent information in a memory device.

An *information state* is an eidostate whose elements are all record states, and the set of such eidostates is denoted I. All information states are uniform eidostates. An *information process* is one that is equivalent to a process $\langle I,J\rangle$, where I,J∈I. (Of course, information processes also include processes of the form $\langle I+x,J+x\rangle$ for a non-record state x∈S, as well as more complex combinations of record and non-record states.) Roughly speaking, an information process is a kind of computation performed on information states.

It is convenient at this point to define a *bit state* (denoted $I_{b}$) as an information state containing exactly two record states. That is, $I_{b}=\left\{r_{0},r_{1}\right\}$. A *bit process* is an information process of the form $\Theta_{b}=\langle r,I_{b}\rangle$ for some r∈R—that is, a process by which a bit state is created from a single record state.

**Figure 3:**
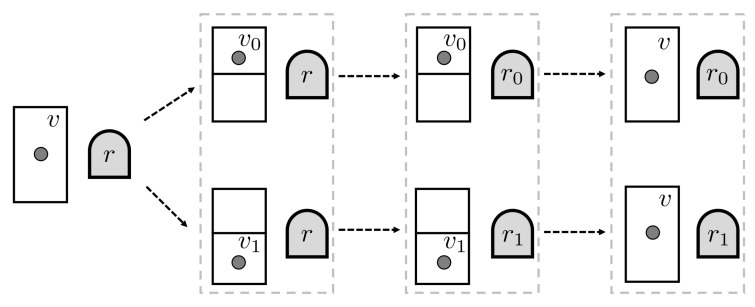
Maxwell’s demon interacting with a one-particle gas, illustrating a bit process $\langle r,\{r_{0},r_{1}\}\rangle$.

We can illustrate a bit process in a thermodynamics context by considering a thought-experiment involving Maxwell’s demon. We imagine that the demon operates on a one-particle gas confined to a volume, a situation described by gas state *v* (see Figure 3). The demon, whose memory initially has a record state *r*, inserts a partition into the container, dividing the volume in two halves labeled 0 and 1. The gas molecule is certainly in one sub-volume or the other. The demon then records in memory which half the particle occupies. Finally, the partition is removed and the particle wanders freely around the whole volume of the container. In thermodynamic terms, the gas state relaxes to the original state *v*. The overall process establishes the following relations:

(14) $$v+r\to \left\{v_{0},v_{1}\right\}+r\to \left\{v_{0}+r_{0},v_{1}+r_{1}\right\}\to v+\left\{r_{0},r_{1}\right\},$$

and so $r\to \{r_{0},r_{1}\}$. In our example, the bit process $\Theta_{b}=\langle r,I_{b}\rangle$ is natural one.

However, we do not yet know that there actually *are* information states and information processes in our theory. We address this by a new axiom.

**Axiom** **5.** Information: There exist a bit state and a possible bit process.

Axiom 5 has a wealth of consequences. Since an information state exists, record states necessarily also exist. There are infinitely many record states, since for any r∈R we also have distinct states r+r,r+(r+r),…,nr,…, all in R. We may have information states in I that contain arbitrarily many record states, because $nI_{b}$ contains $2^{n}$ elements. Furthermore, since every nonempty subset of an information state is also an information state, for any integer k≥1 there exists I∈I so that #(I)=k.

Any two bit states can be reversibly transformed into one another. Consider $I_{b}=\left\{r_{0},r_{1}\right\}$ and $I_{b}^{\prime }=\left\{s_{0},s_{1}\right\}$. Since $r_{0}\to s_{0}$ and $r_{1}\to s_{1}$, it follows by Axiom 4 that $I_{b}\to I_{b}^{\prime }$ (and hence $I_{b}↔I_{b}^{\prime }$.)

If any bit process is possible, then every bit process is possible. Furthermore, every such process is of the natural irreversible type. To see why, consider the bit state $I_{b}=\left\{r_{0},r_{1}\right\}$ and suppose $I_{b}\to r$ for a record state *r*. Since $r\to r_{0}$, this implies that $\left\{r_{0},r_{1}\right\}\to r_{0}$, which is a violation of Axiom 3. Therefore, it must be that $r\to I_{b}$ but $I_{b}↛r$; and this is true for any choice of *r* and $I_{b}$.

Now, we can prove that every information process is possible, and that the → relation is determined solely by the relative sizes of the initial and final information states.

**Theorem** **3.** Suppose I,J∈I. Then, I→J if and only if #(I)≤#(J).

**Proof.**  To begin with, we can see that #(I)=#(J) implies I→J. This is because we can write $I=\{r_{1},\ldots ,r_{n}\}$ and $J=\{s_{1},\ldots ,s_{n}\}$. Since $r_{k}\to s_{k}$ for every k=1,…,n, the finite extension of Axiom 4 tells us that I→J.

Now, imagine that #(I)>#(J). There exists a proper subset $I^{\prime }$ of *I* so that $\#\left(I^{\prime }\right)=\#\left(J\right)$, and thus $J\to I^{\prime }$. If it happened that I→J, it would follow that $I\to I^{\prime }$, a contradiction of Axiom 4. Hence, #(I)>#(J) implies I↛J.

It remains to show that if #(I)<#(J), it must be that I→J. As a first case, suppose that #(I)=n and #(J)=n+1. Letting $I=\{r_{1},\ldots ,r_{n}\}$ and $J=\{s_{1},\ldots ,s_{n},s_{n+1}\}$, we note that $r_{1}\to s_{1},r_{2}\to s_{2},\ldots ,r_{n}\to \left\{s_{n},s_{n+1}\right\}$ (the last being a bit process. It follows that I→J.

We now proceed inductively. Given #(J)=#(I)+n, we can imagine a sequence of information states $K_{m}$ with successive numbers of elements between #(I) and #(J), so that $\#\left(K_{m}\right)=\#\left(I\right)+m$. From what we have already proved,

(15) $$I\to K_{1}\to \cdots \to K_{n-1}\to J,$$

and by transitivity of the → relation we may conclude that I→J. Therefore, I→J if and only if #(I)≤#(J). ☐

Intuitively, the greater the number of distinct record states in an information state, the more information it represents. Thus, under our axioms, a natural information process may maintain or increase the amount of information, but never decrease it.

We can sharpen this intuition considerably. Let $P_{I}$ be the set of information processes, which is closed under addition and negation, and in which every process is possible. We can define a function I on $P_{I}$ as follows: For $\Gamma =\left[\langle I,J\rangle \right]\in P_{I}$,

(16) $$I\left(\Gamma \right)=\log\left(\frac{\#(J)}{\#(I)}\right)=\log\#\left(J\right)-\log\#\left(I\right).$$

The logarithm function guarantees the additivity of I when two processes are combined, and the sign of I exactly determines whether I→J. Thus, I is an irreversibility function on $P_{I}$, as the notation suggests. This function is unique up to a positive constant factor—i.e., the choice of logarithm base. If we choose to use base-2 logarithms, so that a bit process has $I(\Theta_{b})=1$, then I is uniquely determined.

The irreversibility function on information processes can be expressed in terms of a function on information states. Suppose we have a collection of eidostates K⊆E that is closed under the + operation. With Giles, we define a *quasi-entropy* on K to be a real-valued function S such that, for A,B∈K:

1. S(A+B)=S(A)+S(B).
2. If $\langle A,B\rangle$ is natural irreversible, then S(A)<S(B).
3. If $\langle A,B\rangle$ is reversible, then S(A)=S(B).

(A full “entropy” function satisfies one additional requirement, which we will address in Section 8 below.) Given a quasi-entropy S, we can derive an irreversibility function I on possible K-processes by $I\left(\langle A,B\rangle \right)=S\left(B\right)-S\left(A\right)$.

Obviously, S(I)=log#(I) is a quasi-entropy function on I that yields the irreversibility function on $P_{I}$. We recognize it as the Hartley–Shannon entropy of an information source with #(I) possible outputs [16]. In fact, this is the only possible quasi-entropy on I. Since every information process is possible, two different quasi-entropy functions S and $S^{\prime }$ can only differ by an additive constant. However, since I+r↔I for I∈I and r∈R, we know that S(r)=0 for any quasi-entropy. Therefore, S(I)=log#(I) is the unique quasi-entropy function for information states. The quasi-entropy of a bit state is $S(I_{b})=1$.

## 6. Demons

Maxwell’s demon accomplishes changes in thermodynamic states by acquiring and using information. For example, a demon that operates a trapdoor between two containers of gas can arrange for all of the gas molecules to end up in one of the containers, “compressing” the gas without work. As we have seen, it is also possible to imagine a reversible demon, which acquires and manipulates information in a completely reversible way. If such a demon produces a transformation from state *x* to state *y* by acquiring *k* bits of information in its memory, it can accomplish the reverse transformation (from *y* to *x*) while erasing *k* bits from its memory.

Maxwell’s demon is a key concept in axiomatic information thermodynamics, and we introduce a new axiom to describe “demonic” processes.

**Axiom** **6.** *Demons: Suppose a,b∈S and J∈I such that a→b+J.*

*(a)*  There exists I∈I such that b→a+I.
*(b)*  For any I∈I, either a→b+I or b+I→a.

In Part (a), we assert that what one demon can do (transforming *a* to *b* by acquiring information in *J*), another demon can undo (transforming *b* to *a* by acquiring information in *I*). Part (b) envisions a reversible demon. Any amount of information in *I* is either large enough that we can turn *a* to *b* by acquiring *I*, or small enough that we can erase the information by turning *b* to *a*.

A process $\langle A,B\rangle$ is said to be *demonically possible* if one of two equivalent conditions hold:

- There exists an information state J∈I such that either A→B+J or B→A+J.
- There exists an information process $\langle I,J\rangle \in P_{I}$ such that $\langle A,B\rangle +\langle I,J\rangle$ is possible; that is, either A+I→B+J or B+J→A+I.

It is not hard to see that these are equivalent. Suppose we have J∈I such that A→B+J. Then, for any I∈I, A+I→B+(I+J). Conversely, we note that A→A+I for any I∈I. Thus, if A+I→B+J then A→B+J as well.

If a process is possible, then it is also demonically possible, since if A→B it is also true that A+I→B+I. For singleton processes in $P_{S}$, moreover, the converse is also true. Suppose a,b∈S and $\langle a,b\rangle$ is demonically possible. Then, there exists I∈I such that either a→b+I or b→a+I. Either way, either trivially or by an application of Axiom 6, there must be J∈I so that a→b+J. A single record state r∈R is a singleton information state in I. Thus, by Axiom 6 it must be that either a→b+r or b+r→a. Since b+r↔b, we find that a⇌b, and so $\langle a,b\rangle$ is possible.

A singleton process is demonically possible if and only if it is possible. This means that we can use processes involving demons to understand processes that do not. In fact, we can use Axiom 6 to prove a highly significant fact about the → relation on singleton eidostates.

**Theorem** **4.** Suppose a,b,c∈S. If $\langle a,b\rangle$ and $\langle a,c\rangle$ are possible, then $\langle b,c\rangle$ is possible.

**Proof.**  First, we note the general fact that, if $\langle x,y\rangle$ is a possible singleton process, then there exist I,J∈I so that x→y+I and y→x+J.

Given our hypothesis, therefore, there must be I,J∈I such that b→a+I and a→c+J. Then

(17) b→a+I→c+(I+J).

That is, $\langle b,c\rangle$ is demonically possible, and hence possible. ☐

This fact is so fundamental that Giles made it an axiom in his theory. For us, it is a straightforward consequence of the axiom about processes involving demons. It tells us that the set of singleton states S is partitioned into equivalence classes, within each of which all states are related by ⇌.

This statement is more primitive than, but closely related to, a well-known principle called the Comparison Hypothesis. The Comparison Hypothesis deals with a state relation called *adiabatic accessibility* (denoted ≺) which is definable in Giles’s theory (and ours) but is taken as an undefined relation in some other axiomatic developments. According to the Comparison Hypothesis, if *X* and *Y* are states in a given thermodynamic space, either X≺Y or Y≺X. Lieb and Yngvason, for instance, show that the Comparison Hypothesis can emerge as a consequence of certain axioms for thermodynamic states, spaces, and equilibrium [2,3].

Theorem 4 also sheds light on our axiom about conditional processes, Axiom 4. In Part (a) of this axiom, we suppose that A→b for some A∈E and b∈S. The axiom itself allows us to infer that a→b for every a∈A. However, Theorem 4 now implies that, for every $a,a^{\prime }\in A$, either $a\to a^{\prime }$ or $a^{\prime }\to a$. In other words, Part (a) of Axiom 4, similar to Part (b) of the same axiom, only applies to uniform eidostates.

Finally, we introduce one further “demonic” axiom.

**Axiom** **7.** Stability: Suppose A,B∈E and J∈I. If nA→nB+J for arbitrarily large values of n, then A→B.

According to the Stability Axiom, if a demon can transform arbitrarily many copies of eidostate *A* into arbitrarily many copies of *B* while acquiring a bounded amount of information, then we may say that A→B. This can be viewed as a kind of “asymptotic regularization” of the → relation. The form of the Stability Axiom that we have chosen is a particularly simple one, and it suffices for our purposes in this paper. However, more sophisticated axiomatic developments might require a refinement of the axiom. Compare, for instance, Axiom 2.1.3 in Giles to its refinement in Axiom 7.2.1 [1].

To illustrate the use of the Stability Axiom, suppose that A,B∈E and I∈I such that A+I→B+I. Our axioms do not provide a “cancellation law” for information states, so we cannot immediately conclude that A→B. However, we can show that nA→nB+I for all positive integers *n*. The case n=1 holds since A→A+I→B+I. Now, we proceed inductively, assuming that nA→nB+I for some *n*. Then,

(18) $$\begin{array}{ccc} (n+1)A & \to & nA+A\\ & \to & (nB+I)+A\\ & \to & nB+(A+I)\\ & \to & nB+(B+I)\to (n+1)B+I. \end{array}$$

Thus, nA→nB+I for arbitrarily large (and indeed all) values of *n*. By the Stability Axiom, we see that A→B. Thus, there is after all a general cancellation law information states that appear on both sides of the → relation.

## 7. Irreversibility for Singleton Processes

From our two “demonic” axioms (Axioms 6 and 7), we can use the properties of information states to derive an irreversibility function on singleton processes. Let ${\hat{P}}_{S}$ denote the set of possible singleton processes. This is a subgroup of the Abelian group $P_{S}$. Thus, if we can find an irreversibility function I on ${\hat{P}}_{S}$, we will be able to extend it to all of $P_{S}$.

We begin by proving a useful fact about possible singleton processes:

**Theorem** **5.** Suppose a,b∈S so that $\alpha =\langle a,b\rangle$ is possible. Then, for any integers n,m≥0, either $a+mI_{b}\to b+nI_{b}$ or $b+nI_{b}\to a+mI_{b}$.

**Proof.**  If m=n, the result is easy. Suppose that n>m, so that n=m+k for positive integer *k*. By Axiom 6, either $a\to b+kI_{b}$ or $b+kI_{b}\to a$. We can then append the information state $mI_{b}$ to both sides and rearrange the components. The argument for m>n is exactly similar. ☐

This fact has a corollary that we may state using the → relation on processes. Suppose $\alpha =\langle a,b\rangle$ is a possible singleton process, and let q,p be integers with q>0. Then, either $q\alpha \to p\Theta_{b}$ or $q\alpha \leftarrow p\Theta_{b}$ (so that $q\alpha -p\Theta_{b}$ is either natural or antinatural) for the bit process $\Theta_{b}=\langle r,I_{b}\rangle$.

Given α, therefore, we can define two sets of rational numbers:

(19) $$L_{\alpha }=\left\{p/q:q\alpha \to p\Theta_{b}\right\}\;U_{\alpha }=\left\{p/q:q\alpha \leftarrow p\Theta_{b}\right\}$$

where q>0. Both sets are nonempty and every rational number is in at least one of these sets. Furthermore, if $p/q\in U_{\alpha }$ and $p^{\prime }/q^{\prime }\in L_{\alpha }$, we have that $q\alpha \leftarrow p\Theta_{b}$ and $q^{\prime }\alpha \to p^{\prime }\Theta_{b}$, and so

(20) $$pq^{\prime }\Theta_{b}\to qq^{\prime }\alpha \to p^{\prime }q\Theta_{b}.$$

Hence, $\left(pq^{\prime }-p^{\prime }q\right)\Theta_{b}\to 0$. Since $\Theta_{b}$ is itself a natural irreversible process, it follows that $pq^{\prime }-p^{\prime }q\geq 0$, and so

(21) $$\frac{p}{q}\geq \frac{p^{\prime }}{q^{\prime }}.$$

Every element of $U_{\alpha }$ is an upper bound for $L_{\alpha }$. It follows that $U_{\alpha }$ and $L_{\alpha }$ form a Dedekind cut of the rationals, which leads us to the following important result.

**Theorem** **6.** For $\alpha \in {\hat{P}}_{S}$, define $I\left(\alpha \right)=\inf U_{\alpha }=\sup L_{\alpha }$. Then, I is an irreversibility function on ${\hat{P}}_{S}$.

**Proof.**  First, we must show that I is additive. Suppose $\alpha ,\beta \in {\hat{P}}_{S}$. If $p/q\in U_{\alpha }$ and $p^{\prime }/q\in U_{\beta }$, then $q\left(\alpha +\beta \right)\to \left(p+p^{\prime }\right)\Theta_{b}$, and so $\left(p+p^{\prime }\right)/q\in U_{\alpha +\beta }$. It follows that I(α+β)≤I(α)+I(β). The corresponding argument involving $L_{\alpha }$ and $L_{\beta }$ proves that I(α+β)≥I(α)+I(β). Thus, I must be additive.

Next, we must show that the value of I(α) tells us the type of the process α. If I(α)>0, then $0\in L_{\alpha }$ but $0\notin U_{\alpha }$, and so α→0 but α↚0. That is, α is natural irreversible. Likewise, if I(α)<0, then $0\notin L_{\alpha }$ but $0\in U_{\alpha }$, from which we find that α must be antinatural irreversible. Finally, if I(α)=0, we find that $q\alpha -\Theta_{b}\to 0$ and $q\alpha +\Theta_{b}\leftarrow 0$ for arbitrarily large values of *q*. From Axiom 7, we may conclude that α↔0, and so α is reversible. ☐

Notice that we have arrived at an irreversibility function for possible singleton processes—those most analogous to the ordinary processes in Giles or any text on classical thermodynamics—from axioms about information and processes involving demons (Axioms 5–7). In our view, such ideas are not “extras” to be appended onto a thermodynamic theory, but are instead central concepts throughout. In ordinary thermodynamics, the possibility of a reversible heat engine can have implications for processes that do not involve any heat engines at all. In the information thermodynamics whose axiomatic foundations we are exploring, the possibility of a Maxwell’s demon has implications even for situations in which no demon acts.

We now have irreversibility functions for both information processes and singleton processes. These are closely related. In fact, it is possible to prove the following general result:

**Theorem** **7.** If $\alpha \in {\hat{P}}_{S}$ and $\Gamma \in P_{I}$, then the combined process α+Γ is natural if and only if I(α)+I(Γ)≥0.

Since the set ${\hat{P}}_{S}$ of possible singleton processes is a subgroup of the Abelian group $P_{S}$ of all singleton processes, we can extend the additive irreversiblity function I to all of $P_{S}$. Though I is unique on the possible set ${\hat{P}}_{S}$, its extension to $P_{S}$ is generally not unique.

## 8. Components of Content and Entropy

Our axiomatic theory of information thermodynamics is fundamentally about the set of eidostates E. However, the part of that theory dealing with the set S of singleton eidostates includes many of the concepts and results of ordinary axiomatic thermodynamics [1]. We have a group of singleton processes $P_{S}$ containing a subgroup ${\hat{P}}_{S}$ of possible processes, and we have constructed an irreversibility function I on ${\hat{P}}_{S}$ that may be extended to all of $P_{S}$. From these we can establish several facts.

- We can construct *components of content*, which are the abstract versions of conserved quantities. A component of content *Q* is an additive function on S such that, if the singleton process $\langle a,b\rangle$ is possible, then Q(a)=Q(b). (In conventional thermodynamics, components of content include energy, particle number, etc.)
- We can find a *sufficient set* of components of content. The singleton process $\langle a,b\rangle$ is possible if and only if Q(a)=Q(b) for all *Q* in the sufficient set.
- We can use I to define a *quasi-entropy* S on S as follows: $S\left(a\right)=I\left(\langle a,2a\rangle \right)$. This is an additive function on states in S such that $I\left(\langle a,b\rangle \right)=S\left(b\right)-S\left(a\right)$.

Because the extension of the irreversibility function I from ${\hat{P}}_{S}$ to all of $P_{S}$ is not unique, the quasi-entropy S is not unique either. How could various quasi-entropies differ? Suppose $I_{1}$ and $I_{2}$ are two different extensions of the same original I, leading to two quasi-entropy functions $S_{1}$ and $S_{2}$ on S. Then, the difference $Q=S_{1}-S_{2}$ is a component of content. That is, if $\langle a,b\rangle$ is possible,

(22) $$\begin{array}{ccc} Q(b)-Q(a) & = & S_{1}\left(b\right)-S_{2}\left(b\right)-S_{1}\left(a\right)+S_{2}\left(a\right)\\ & = & I_{1}\left(\langle a,b\rangle \right)-I_{2}\left(\langle a,b\rangle \right)\\ & = & 0, \end{array}$$

since $I_{1}$ and $I_{2}$ agree on ${\hat{P}}_{S}$, which contains $\langle a,b\rangle$.

Another idea that we can inherit without alteration is the concept of a *mechanical state*. A mechanical state is a singleton state that reversibly stores one or more components of content, in much the same way that we can store energy reversibly as the work done to lift or lower a weight. The mechanical state in this example is the height of the weight. In Giles’s theory [1], mechanical states are the subject of an axiom, which we also adopt:

**Axiom** **8.** *Mechanical states: There exists a subset M⊆S of* mechanical states *such that:*

*(a)*  If l,m∈M, then l+m∈M.
*(b)*  For l,m∈M, if l→m then m→l.

Nothing in this axiom asserts the actual *existence* of any mechanical state. It might be that M=∅. Furthermore, the choice of the designated set M is not determined solely by the → relations among the states. For instance, the set R of record states might be included in M, or not. This explains why the introduction of mechanical states must be phrased as an axiom, rather than a definition: a complete specification of the system must include the choice of which set is to be designated as M. Whatever choice is made for M, the set $P_{M}$ of *mechanical processes* (i.e., those equivalent to $\langle l,m\rangle$ for l,m∈M) will form a subgroup of $P_{S}$.

A mechanical state may “reversibly store” a component of content *Q*, but it need not be true that every *Q* can be stored like this. We say that a component of content *Q* is *non-mechanical* if Q(m)=0 for all m∈M. For example, we might store energy by lifting or lowering a weight, but we cannot store particle number in this way.

Once we have mechanical states and processes, we can give a new classification of processes. A process Γ∈P is said to be *adiabatically natural* (*possible*, *reversible*, *antinatural*) if there exists a mechanical process $\mu \in P_{M}$ such that Γ+μ is natural (possible, reversible, antinatural). We can also define the “adiabatic accessibility” relation for states in S, as mentioned in Section 6: a≺b whenever the process $\langle a,b\rangle$ is adiabatically natural.

The set M of mechanical states allows us to refine the idea of a quasi-entropy into an *entropy*, which is a quasi-entropy S that takes the value S(m)=0 for any mechanical state *m*. Such a function is guaranteed to exist. We end up with a characterization theorem, identical to a result of Giles [1], that summarizes the general thermodynamics of singleton eidostates in our axiomatic theory.

**Theorem** **8.** *There exist an entropy function S and a set of components of content Q on S with the following properties:*

*(a)*  For any a,b∈S, S(a+b)=S(a)+S(b).
*(b)*  For any a,b∈S and component of content Q, Q(a+b)=Q(a)+Q(b).
*(c)*  For any a,b∈S, a→b if and only if S(a)≤S(b) and Q(a)=Q(b) for every component of content Q.
*(d)*  S(m)=0 for all m∈M.

An entropy function is not unique. Two entropy functions may differ by a non-mechanical component of content.

The entropy function on S is related to the information entropy function we found for information states in I. Suppose we have a,b∈S and I,J∈I. Then, Theorem 7 tells us that a+I→b+J only if

(23) S(a)+log#(I)≤S(b)+log#(J).

Let $E_{SI}$ represent the set of eidostates that are similar to a singleton state combined with an information state. Then, S(a+I)=S(a)+log#(I) is an entropy function on $E_{SI}$. In the next section, we will extend the domain of the entropy function even further, to the set U of all uniform eidostates.

## 9. State Equivalence

**Figure 4:**
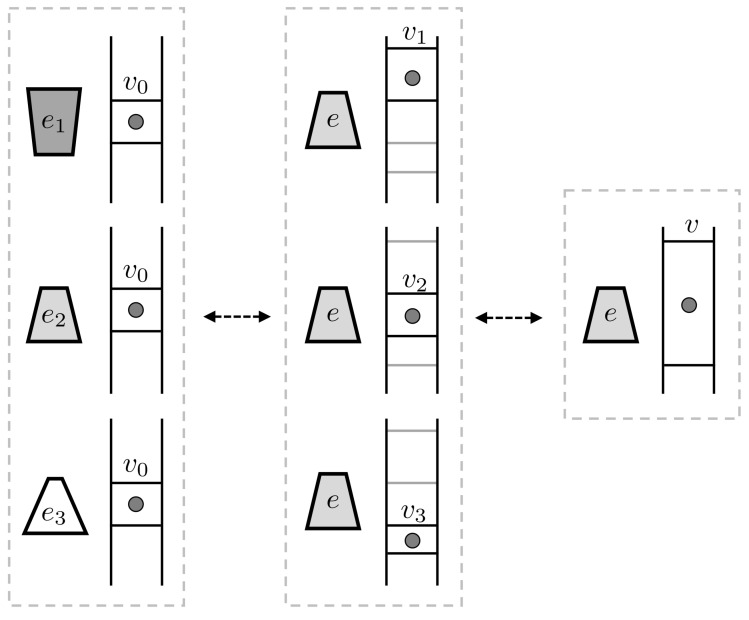
A state equivalence thought-experiment involving states of an arbitrary thermodynamic system and a one-particle gas.

Consider a thought-experiment (illustrated in Figure 4) in which a one-particle gas starts out in a volume $v_{0}$ and a second thermodynamic system starts out in one of three states $e_{1}$, $e_{2}$ or $e_{3}$. We assume that all conserved quantities are the same for these three states, but they may differ in entropy. We can formally describe the overall situation by the eidostate $E+v_{0}$, where $E=\{e_{1},e_{2},e_{3}\}$ is uniform.

We can reversibly transform each of the $e_{k}$ states to the same state *e*, compensating for the various changes in entropy by expanding or contracting the volume occupied by the gas. That is, we can have $v_{0}+e_{k}↔v_{k}+e$ for adjacent but non-overlapping volumes $v_{k}$. Axiom 4 indicates that we can write $E+v_{0}↔e+V$, where $V=\{v_{1},v_{2},v_{3}\}$.

Now, we note that *V* can itself be reversibly transformed into a singleton eidostate *v*. A gas molecule in one of the sub-volumes (eidostate *V*) can be turned into a molecule in the whole volume (eidostate *e*) by removing internal partitions between the sub-volumes; and when we re-insert these partitions the particle is once again in just one of sub-volumes. Thus, V↔v. To summarize, we have

(24) $$E+v_{0}↔e+v.$$

The uniform eidostate *E*, taken together with the gas state $v_{0}$, can be reversibly transformed into the singleton state v+e. We call this a *state equivalence* for the uniform eidostate *E*. By choosing the state *e* properly (say, by letting $e=e_{k}$ for some *k*), we can also guarantee that the volume *v* is larger than $v_{0}$, so that $v_{0}\to v$ by free expansion.

This discussion motivates our final axiom, which states that this type of reversible transformation is always possible for a uniform eidostate.

**Axiom** **9.** State equivalence: If E is a uniform eidostate then there exist states e,x,y∈S such that x→y and E+x↔e+y.

Axiom 9 closes a number of gaps in our theory. For example, our previous axioms (Axioms 1–8) do not by themselves guarantee than *any* state in S has a nonzero entropy. With the new axiom, however, we can prove that such states exist. The bit state $I_{b}$, which is uniform, has some state equivalence given by $I_{b}+x↔e+y$. Since the eidostates on each side of this relation are in $E_{SI}$, we can determine the entropies on each side. We find that

(25) 1+S(x)=S(e)+S(y).

It follows that at least one of the states e,x,y must have S≠0.

State equivalence also allows us to define the entropy of any uniform eidostate E∈U. If E+x↔e+y then we let

(26) S(E)=S(e)+S(y)−S(x).

We must first establish that this expression is well-defined. If we have two state equivalences for the same *E*, so that E+x↔e+y and $E+x^{\prime }↔e^{\prime }+y^{\prime }$, then

(27) $$\left(e+y\right)+x^{\prime }↔E+\left(x+x^{\prime }\right)↔\left(e^{\prime }+y^{\prime }\right)+x,$$

from which it follows that

(28) $$S\left(e\right)+S\left(y\right)-S\left(x\right)=S\left(e^{\prime }\right)+S\left(y^{\prime }\right)-S\left(x^{\prime }\right).$$

Thus, our definition for S(E) does not depend on our choice of state equivalence for *E*.

Is S an entropy function on U? It is straightforward to show that S is additive on U and that S(m)=0 for any mechanical state *m*. It remains to show that, for any E,F∈U with $\langle E,F\rangle$ possible, E→F if and only if S(E)≤S(F). We will use the state equivalences E+x↔e+y and F+w↔f+z.

Suppose first that E→F. Then, E+(x+w)→F+(x+w) and so

(29) (e+y)+w↔(E+x)+w→(F+w)+x↔(f+z)+x.

From this, it follows that

(30) S(e)+S(y)−S(x)≤S(f)+S(z)−S(w),

and hence S(E)≤S(F).

We can actually extract one more fact from this argument. If we assume that $\langle E,F\rangle$ is possible, it must also be true that $\langle (e+y)+w,(f+z)+x\rangle$ is a possible singleton process. If we now suppose that S(E)≤S(F), we know that S((e+y)+w)≤S((f+z)+x) and thus

(31) E+(x+w)↔(e+y)+w→(f+z)+x↔F+(x+w).

Therefore, E→F, as desired.

We have extended the entropy S to uniform eidostates. It is even easier to extend any component of content function *Q* to these states. If E∈U, then any $e_{1},e_{2}\in E$ must have $Q\left(e_{1}\right)=Q\left(e_{2}\right)$, since $e_{1}⇌e_{2}$. Thus, we can define $Q\left(E\right)=Q\left(e_{k}\right)$ for any $e_{k}\in E$. This is additive because the elements of E+F are combinations $e_{k}+f_{j}$ of states in *E* and *F*. Furthermore, suppose we have a state equivalence E+x↔e+y. Since we assume x→y in a state equivalence, Q(x)=Q(y). By the conditional process axiom (Axiom 4) we know that $e_{k}+x\to e+y$ for any $e_{k}\in E$. It follows that $Q\left(e\right)=Q\left(e_{k}\right)=Q\left(E\right)$.

Now, let $E_{1}$ and $E_{2}$ be uniform eidostates with state equivalences $E_{k}+x_{k}↔e_{k}+y_{k}$. Suppose further that $Q\left(E_{1}\right)=Q\left(E_{2}\right)$ for every component of content *Q*. We know that $Q\left(x_{1}\right)=Q\left(y_{1}\right)$, $Q\left(x_{2}\right)=Q\left(y_{2}\right)$ and $Q\left(e_{1}\right)=Q\left(e_{2}\right)$ for every component of content. Thus

(32) $$E_{1}+\left(x_{1}+x_{2}\right)↔\left(e_{1}+y_{1}\right)+x_{2}⇌\left(e_{2}+y_{2}\right)+x_{1}↔E_{2}+\left(x_{1}+x_{2}\right).$$

It follows that $E_{1}⇌E_{2}$, i.e., that $\langle E_{1},E_{2}\rangle$ is a possible eidostate process.

We have therefore extended Theorem 8 to all uniform eidostates. We state the new result here.

**Theorem** **9** (Uniform eidostate thermodynamics)**.** *There exist an entropy function S and a set of components of content Q on U with the following properties:*

*(a)*  For any E,F∈U, S(E+F)=S(E)+S(F).
*(b)*  For any E,F∈U and component of content Q, Q(E+F)=Q(E)+Q(F).
*(c)*  For any E,F∈U, E→F if and only if S(E)≤S(F) and Q(E)=Q(F) for every component of content Q.
*(d)*  S(m)=0 for all m∈M.

The set U of uniform eidostates includes the singleton states in S, the information states in I, all combinations of these, and perhaps many other states as well. (Non-uniform eidostates in E *might* exist, as we will see in the model we discuss in Section 12, but their existence cannot be proved from our axioms.) The type of every process involving uniform eidostates can be determined by a single entropy function (which must not decrease) and a set of components of content (which must be conserved).

## 10. Entropy for Uniform Eidostates

We have extended the entropy function from singleton states and information states to all uniform eidostates. It turns out that this extension is unique. The following theorem and its corollaries actually allow us to compute the entropy of any E∈U from the entropies of the states contained in *E*.

**Theorem** **10.** *Suppose E∈U is a disjoint union of uniform eidostates $E_{1}$ and $E_{2}$. Then*

(33) $$S\left(E\right)=S\left(E_{1}\cup E_{2}\right)=\log\left(2^{S(E_{1})}+2^{S(E_{2})}\right).$$

**Proof.**  *E* and $E_{k}$ (k=1,2) all have equal components of content. Define

(34) $$\Delta \left(E_{1},E_{2}\right)=S\left(E\right)-\log\left(2^{S(E_{1})}+2^{S(E_{2})}\right).$$

Note that, if we replace $E_{k}$ by $E_{k}^{\prime }=E_{k}+I$ for I∈I, then these new eidostates are still disjoint and

(35) $$E^{\prime }=E_{1}^{\prime }\cup E_{2}^{\prime }=\left(E_{1}+I\right)\cup \left(E_{2}+I\right)=\left(E+I\right).$$

These eidostates have the same components of content as the original *E*. Furthermore,

(36) $$\begin{array}{ccc} \Delta (E_{1}^{\prime },E_{2}^{\prime }) & = & \Delta (E_{1}+I,E_{2}+I)\\ & = & S\left(E+I\right)-\log\left(2^{S(E_{1}+I)}+2^{S(E_{2}+I)}\right)\\ & = & S\left(E\right)+\log\#\left(I\right)-\log\left(2^{\log\#(I)}\left(2^{S(E_{1})}+2^{S(E_{2})}\right)\right)\\ & = & \Delta (E_{1},E_{2}). \end{array}$$

We can find a uniform eidostate $E_{0}$ with the same components of content such that $S_{0}=S\left(E_{0}\right)$ is less than or equal to $S(E_{1})$, $S(E_{2})$ and S(E). (It suffices to pick $E_{0}$ to be the state of smallest entropy among $E_{1}$, $E_{2}$ and *E*.) Then, there exist integers $m_{k}\geq 1$ such that

- There are disjoint information states $J_{k}$ containing $m_{k}$ record states.
- There are disjoint information states $J_{k}^{*}$ containing $m_{k}+1$ record states.
- $J=J_{1}\cup J_{2}$ and $J^{*}=J_{1}^{*}\cup J_{2}^{*}$ have $m_{1}+m_{2}$ and $m_{1}+m_{2}+2$ record states, respectively.
- We have
  (37) $$S_{0}+\log m_{k}\leq S\left(E_{k}\right)<S_{0}+\log\left(m_{k}+1\right).$$
  That is, we choose $m_{k}$ so that $S\left(E_{k}\right)-S_{0}\geq 0$ is between $\log(m_{k})$ and $\log(m_{k}+1)$. To put it more simply, $m_{k}=\left\lfloor 2^{S\left(E_{k}\right)-S_{0}}\right\rfloor$.

Once we have $m_{k}$, we can write that

(38) $$2^{S_{0}}\cdot m_{k}\leq 2^{S(E_{k})}<2^{S_{0}}\cdot \left(m_{k}+1\right).$$

Adding these inequalities for k=1,2 and taking the logarithm yields

(39) $$S_{0}+\log\left(m_{1}+m_{2}\right)\leq \log\left(2^{S(E_{1})}+2^{S(E_{2})}\right)<S_{0}+\log\left(m_{1}+m_{2}+2\right).$$

How far apart are the two ends of this chain of inequalities? Here, is a useful fact about base-2 logarithms: If n≥1, then log(n+2)<log(n)+2/n. This implies

(40) $$S_{0}+\log\left(m_{1}+m_{2}\right)\leq \log\left(2^{S(E_{1})}+2^{S(E_{2})}\right)<S_{0}+\log\left(m_{1}+m_{2}\right)+\frac{2}{m_{1}+m_{2}}.$$

The two ends of the inequality differ by less than $2/(m_{1}+m_{2})$.

We can get another chain of inequalities by applying Axiom 4 about conditional processes. Since all of our uniform eidostates have the same components of content, we know that

(41) $$E_{0}+J_{k}\to E_{k}\to E_{0}+J_{k}^{*}.$$

From the axiom, we can therefore say

(42) $$E_{0}+J\to E\to E_{0}+J^{*}$$

which implies that

(43) $$\begin{array}{ccc} S_{0}+\log\left(m_{1}+m_{2}\right)\leq S\left(E\right) & \leq & S_{0}+\log\left(m_{1}+m_{2}+2\right)\\ & < & S_{0}+\log\left(m_{1}+m_{2}\right)+\frac{2}{m_{1}+m_{2}}. \end{array}$$

We have two quantities that lie in the same interval. Their separation is therefore bounded by the interval width—i.e., less than $2/(m_{1}+m_{2})$. Therefore,

(44) $$\begin{array}{ccc} \left|\Delta (E_{1},E_{2})\right| & = & \left|S\left(E\right)-\log\left(2^{S(E_{1})}+2^{S(E_{2})}\right)\right|\\ & < & \frac{2}{m_{1}+m_{2}}. \end{array}$$

How big are the numbers $m_{k}$? We can make such numbers as large as we like by considering instead the eidostates $E_{k}^{\prime }=E_{k}+I$, where *I* is an information state. As we have seen, $\Delta \left(E_{1}^{\prime },E_{2}^{\prime }\right)=\Delta \left(E_{1},E_{2}\right)$. Given any ϵ>0, we can choose *I* so that

(45) $$m_{k}^{\prime }=\left\lfloor 2^{S\left(E_{k}^{\prime }\right)-S_{0}}\right\rfloor =\left\lfloor \#\left(I\right)\cdot 2^{S\left(E_{k}\right)-S_{0}}\right\rfloor >\frac{1}{\epsilon }.$$

Then, $2/(m_{1}^{\prime }+m_{2}^{\prime })<\epsilon$, and so

(46) $$\left|\Delta (E_{1},E_{2})\right|=\left|\Delta (E_{1}^{\prime },E_{2}^{\prime })\right|<\epsilon .$$

Since this is true for any ϵ>0, we must have $\Delta (E_{1},E_{2})=0$, and so

(47) $$S\left(E\right)=\log\left(2^{S(E_{1})}+2^{S(E_{2})}\right),$$

as desired. ☐

Theorem 10 has a corollary, which we obtain by applying the theorem inductively:

**Theorem** **11.** *If E is a uniform eidostate,*

(48) $$S\left(E\right)=\log\left(\sum_{e_{k}\in E}2^{S(e_{k})}\right).$$

The entropy of any uniform eidostate is a straightforward function of the entropies of the states contained therein. If *E* contains more than one state, we notice that $S\left(E\right)>S\left(e_{k}\right)$ for any $e_{k}\in E$. It follows that $\langle e_{k},E\rangle$ is a natural irreversible process.

To take a simple example of Theorem 11, consider the entropy of an information state. Every record state *r* has S(r)=0. Thus, for I∈I,

(49) $$S\left(I\right)=\log\left(\sum_{r_{k}\in I}2^{0}\right)=\log\#\left(I\right),$$

as we have already seen.

## 11. Probability

An eidostate in E represents a state of knowledge of a thermodynamic agent. It is, as we have said, a simple list of possible states, without any assignment of probabilities to them. If the agent is to use probabilistic reasoning, then it needs to assign conditional probabilities of the form P(A|B) where A,B∈E.

The entropy formula in Theorem 11 allows us to make such an assignment based on the entropy itself, provided the eidostate conditioned upon is uniform. Suppose E∈U and let a∈S. Then, the *entropic probability* of *a* conditioned on *E* is

(50) $$P\left(a|E\right)=\left\{\begin{array}{cc} \frac{2^{S(a)}}{2^{S(E)}}=2^{S(a)-S(E)} & \;a\in E\\ 0 & \;a\notin E \end{array}\right..$$

Clearly, 0≤P(a|E)≤1 and $\sum_{a}P\left(a|E\right)=1$. It is worth noting that, although *E* is “uniform” (in the sense that all a∈E have exactly the same conserved components of content), the probability distribution P(a|E) is *not* uniform, but assigns a higher probability to states of higher entropy.

We can generalize entropic probabilities a bit further. Let *A* be any subset of S (be it an eidostate or not) and E∈U. Then, A∩E is either a uniform eidostate or the empty set ∅. If we formally assign S(∅)=−∞, then both *E* and A∩E have well-defined entropies. Then, we define

(51) $$p\left(A|E\right)=\sum_{a\in A}P\left(a|E\right)=\frac{2^{S(A\cap E)}}{2^{S(E)}}=2^{S(A\cap E)-S(E)}.$$

Obviously, P(E|E)=1. Now, consider two disjoint sets *A* and *B* along with E∈U. The set (A∪B)∩E is a disjoint union of uniform eidostates (or empty sets) A∩E and B∩E. Thus,

(52) $$\begin{array}{ccc} P(A\cup B|E) & = & \frac{2^{S((A\cup B)\cap E)}}{2^{S(E)}}\\ & = & \frac{2^{S(A\cap E)}+2^{S(B\cap E)}}{2^{S(E)}}\\ & = & P(A|E)+P(B|E), \end{array}$$

in accordance with the rules of probability. We also have the usual rule for conditional probabilities. Suppose A,B⊆S and E∈U such that A∩E≠∅. Then

(53) $$P\left(B|A\cap E\right)=\frac{2^{S(B\cap (A\cap E))}}{2^{S(A\cap E)}}=\frac{P(B\cap A|E)}{P(A|E)}.$$

The entropy formula in Theorem 11 and the probability assignment in Equation (50) call to mind familiar ideas from statistical mechanics. According to Boltzmann’s formula, the entropy is S=logΩ, where Ω is (depending on the context) the number (or phase space volume or Hilbert space dimension) of the microstates consistent with macroscopic data about a system. In the microcanonical ensemble, a uniform probability distribution is assigned to these microstates. In an eidostate *E* comprising non-overlapping macrostates states $\left\{e_{1},e_{2},\ldots \right\}$, we would therefore expect $\Omega \left(E\right)=\Omega \left(e_{1}\right)+\Omega \left(e_{2}\right)+\ldots$, and the probability of state $e_{k}$ should be proportional to $\Omega (e_{k})$. However, in our axiomatic system Theorem 11 and Equation (50) do not arise from any assumptions about microstates, but solely from the “phenomenological” → relation among eidostates in E.

Even though the entropy function S is not unique, the entropic probability assignment is unique. Suppose $S_{1}$ and $S_{2}$ are two entropy functions for the same states and processes. Then, as we have seen, the difference $S_{1}-S_{2}$ is a component of content. All of the states within a uniform eidostate *E* (as well as *E* itself) have the same values for all components of content. That is, $S_{1}\left(a\right)-S_{2}\left(a\right)=S_{1}\left(E\right)-S_{2}\left(E\right)$ for any a∈E. Thus,

(54) $$P_{1}\left(a|E\right)=2^{S_{1}\left(a\right)-S_{1}\left(E\right)}=2^{S_{2}\left(a\right)-S_{2}\left(E\right)}=P_{2}\left(a|E\right).$$

(Both probabilities are zero for a∉E, of course.)

The entropic probability assignment is not the only possible probability assignment, but it does have a number of remarkable properties. For instance, suppose *E* and *F* are two uniform eidostates. If we prepare them independently, we have the combined eidostate E+F and the probability of some particular state x+y is

(55) $$P\left(x+y|E+F\right)=\frac{2^{S(x)+S(y)}}{2^{S(E)+S(F)}}=P\left(x|E\right)\;P\left(y|F\right),$$

as we would expect for independent events.

The entropic probability also yields an elegant expression for the entropy S(E) of a uniform eidostate *E*.

**Theorem** **12.** *Suppose E is a uniform eidostate, and P(a|E) is the entropic probability for state a∈E. Then*

(56) $$S\left(E\right)=\langle S(a)\rangle +H\left(\vec{P}\right),$$

*where 〈S(a)〉 is the average state entropy in E and $H(\vec{P})$ is the Shannon entropy of the P(a|E) distribution.*

**Proof.**  We first note that, for any a∈E, logP(a|E)=S(a)−S(E). We rewrite this as S(E)=S(a)−logP(a|E) and take the mean value with respect to the P(a|E) probabilities:

(57) $$\begin{array}{ccc} S(E) & = & \langle S(E)\rangle \\ & = & \sum_{a\in E}P\left(a|E\right)\left(S(a)-\log P(a|E)\right)\\ & = & \sum_{a\in E}P\left(a|E\right)S\left(a\right)-\sum_{a\in E}P\left(a|E\right)\log P\left(a|E\right), \end{array}$$

Therefore, $S\left(E\right)=\langle S(a)\rangle +H\left(\vec{P}\right)$, as desired. ☐

We previously said that the list of possible states in an eidostate represents a kind of information. Theorem 12 puts this intuition on a quantitative footing. The entropy of a uniform eidostate *E* can be decomposed into two parts: the average entropy of the states, and an additional term representing the information contained in the distinction among the possible states. For a singleton state, the entropy is all of the first sort. For a pure information state I∈I, it is all of the second.

In fact, the decomposition itself uniquely picks out the entropic probability assignment. Suppose P(a|E) is the entropic probability of *a* given *E*, and $P^{\prime }\left(a|E\right)$ is some other probability distribution over states in *E*. By Gibbs’s inequality [17],

(58) $$0\leq \sum_{a\in E}P^{\prime }\left(a|E\right)\log\left(\frac{P^{\prime }\left(a|E\right)}{P(a|E)}\right)$$

with equality if and only if $P^{\prime }\left(a|E\right)=P\left(a|E\right)$ for all a∈E. We find that

(59) $$\begin{array}{ccc} 0 & \leq & \sum_{a\in E}P^{\prime }\left(a|E\right)\log\left(\frac{P^{\prime }\left(a|E\right)\;2^{S(E)}}{2^{S(a)}}\right)\\ & = & \sum_{a\in E}P^{\prime }\left(a|E\right)\log P^{\prime }\left(a|E\right)\;\;+\;\;S\left(E\right)\;\;-\;\;\sum_{a\in E}P^{\prime }\left(a|E\right)S\left(a\right) \end{array}$$

and so

(60) $$S\left(E\right)\geq {\langle S(a)\rangle }_{\;\;P^{\prime }}+H\left(\vec{P^{\prime }}\right),$$

with equality if and only if the $P^{\prime }$ distribution is the entropic one.

An agent that employs entropic probabilities will regard the entropy of a uniform eidostate *E* as the sum of two parts, one the average entropy of the possible states and the other the Shannon entropy of the distribution. For an agent that employs some other probability distribution, things will not be so simple. Besides the average state entropy and the Shannon entropy of the distribution, S(E) will include an extra, otherwise unexplained term. Thus, for a given collection of eidostates connected by the → relation, the entropic probability provides a uniquely simple account of the entropy of any uniform eidostate.

It is in this sense we say that the entropic probability “emerges” from the entropy function S, just as that function itself emerges from the → relation among eidostates.

Some further remarks about probability are in order. Every formal basis for probability emphasizes a distinct idea about it. In Kolmogorov’s axioms [18], probability is simply a measure on a sample space. High-measure subsets are more probable. In the Bayesian approach of Cox [19], probability is a rational measure of confidence in a proposition. Propositions in which a rational agent is more confident are also more probable. Laplace’s early discussion [20] is based on symmetry. Symmetrically equivalent events—two different orderings of a shuffled deck of cards, for instance—are equally probable. (Zurek [21] has used a similar principle of “envariance” to discuss the origin of quantum probabilities.) In algorithmic information theory [16], the algorithmic probability of a bit string is related to its complexity. Simpler bit strings are more probable.

In a similar way, entropic probabilities express facts about state transformations. In a uniform eidostate *E*, any two states a,b∈E are related by a possible process. If a→b, then the output state is at least as probable as the input state: P(a|E)≤P(b|E).

## 12. A Model for the Axioms

A model for an axiomatic system may serve several purposes. The existence of a model establishes that the axioms are self-consistent. A model may also demonstrate that the system can describe an actual realistic physical situation. If the axioms have a variety of interesting models, then the axiomatic theory is widely applicable. We may almost say that the entire significance of an axiomatic system lies in the range of models for that system.

Terms that are undefined in an abstract axiomatic system are defined within a model as particular mathematical structures. The axioms of the system are provable properties of those structures. Therefore, a model for axiomatic information thermodynamics must include several elements:

- A set S of states and a collection E of finite nonempty subsets of S to be designated as eidostates.
- A rule for interpreting the combination of states (+) in S.
- A relation → on E.
- A designated set M⊆S of mechanical states (which might be empty).
- Proofs of Axioms 1–9 within the model, including the general properties of →, the existence of record states and information states, etc.

The model will therefore involve specific meanings for S, E, +, → and so forth. It will also yield interpretations of derived concepts and results, such as entropy functions and conserved components of content. In the abstract theory, the combination a+b is simply the Cartesian pair (a,b). This definition may suffice for the model, or the model may have a different interpretation of +. In any case, it must be true in the model that $a+b=a^{\prime }+b^{\prime }$ implies $a=a^{\prime }$ and $b=b^{\prime }$.

**Figure 5:**
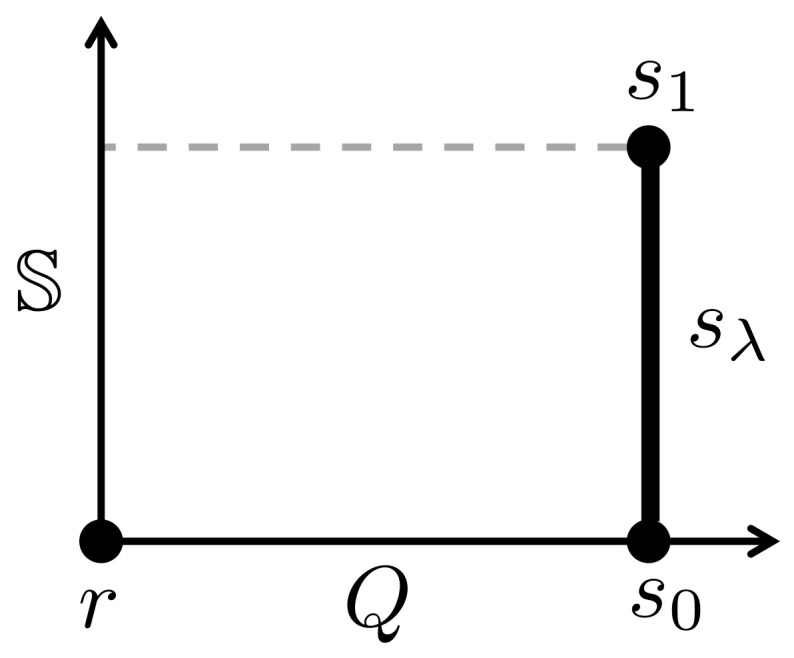
Atomic states in our simple “macrostate” model.

Our first model for axiomatic thermodynamics is based on a set A of “atomic” states, from which all states in S and all eidostates in E are constructed. We assign entropies and components of content to these states, which extend to composite states by additivity. Let us consider a simple but non-trivial example that has just one component of content *Q*. A suitable set A of atomic states is shown in Figure 5. It includes a special state *r* (with S(r)=0 and Q(r)=0) and a continuous set of states $s_{\lambda }$ with $Q(s_{\lambda })=1$ and $S(s_{\lambda })=\lambda$. The parameter λ ranges over the closed interval [0,1].

The set of states S includes everything that can be constructed from A by finite application of the pairing operation. In this way, we can build up a∈S with any non-negative integer value of the component of content Q(a) and any entropy value 0≤S(a)≤Q(a). Indeed, there will typically be many different ways to create given (Q,S) values. To obtain Q(a)=2 and S(a)=1, for example, we might have $a=s_{1}+s_{0},s_{0}+s_{1},\left(s_{1/2}+s_{1/2}\right)+r,\ldots$.

Anticipating somewhat, we call an eidostate *uniform* if all of its elements have the same *Q*-value. We calculate the entropy of a uniform eidostate by applying Theorem 11 to it.

Our model allows any finite nonempty set of states to play the role of an eidostate. Hence, we have both uniform and non-uniform eidostates in E. Each eidostate *A* has a finite Cartesian factorization

(61) $$A=F_{A}\left(E_{1},\ldots ,E_{n}\right).$$

If *A* is uniform, then all of its factors are also uniform. If none of its factors are uniform, we say that *A* is *completely non-uniform*. More generally, we can write down an *NU-decomposition* for any eidostate *A*:

(62) $$A∼N_{A}+U_{A},$$

where $N_{A}$ is a completely non-uniform eidostate and $U_{A}$ is a uniform eidostate. Of course, if *A* itself is either completely non-uniform or uniform, one or the other of these eidostates may be absent from the decomposition. The NU-decomposition is unique up to similarity: If $N_{A}+U_{A}∼N_{A}^{\prime }+U_{A}^{\prime }$ for completely non-uniform *N*s and uniform *U*s, then $N_{A}∼N_{A}^{\prime }$ and $U_{A}∼U_{A}^{\prime }$.

We can now define the → relation on E in our model. If A,B∈E, we first write down NU-decompositions $A∼N_{A}+U_{A}$ and $B∼N_{B}+U_{B}$. We say that A→B provided three conditions hold:

1. Either $N_{A}$ and $N_{B}$ both do not exist, or $N_{A}∼N_{B}$.
2. Either $U_{A}$ and $U_{B}$ both do not exist, or only one exists and its *Q*-value is 0, or both exist and $Q\left(U_{A}\right)=Q\left(U_{B}\right)$.
3. Either $U_{A}$ and $U_{B}$ both do not exist, or only $U_{A}$ exists and $S(U_{A})=0$, or only $U_{B}$ exists and $S(U_{B})\geq 0$, or both exist and $S\left(U_{A}\right)\leq S\left(U_{B}\right)$.

We may call these the *N*-criterion, *Q*-criterion, and S-criterion, and summarize their meaning as follows: A→B provided we can transform *A* to *B* by: (a) rearranging the non-uniform factors; and (b) transforming the uniform factors in a way that conserves *Q* and does not decrease S.

Now, let us examine each of the axioms in turn.

**Axiom** **1** The basic properties of eidostates follow by construction.
**Axiom** **2** Part (a) holds because A∼B implies that *A* and *B* can have the same NU-decomposition. Part (b) holds because similarity, equality (for *Q*) and inequality (for S) are all transitive. Parts (c) and (d) make use of the general facts that $N_{A+B}∼N_{A}+N_{B}$ and $U_{A+B}∼U_{A}+U_{B}$.
**Axiom** **3** If $N_{A}≁N_{B}$, then A↛B. If $N_{A}∼N_{B}$, then it must be true that $U_{B}⊊U_{A}$, and so $S\left(U_{A}\right)>S\left(U_{B}\right)$. The S-criterion fails, so A↛B in this case as well.
**Axiom** **4** For Part (a), we note that *A* must be uniform, and so $A^{\prime }\subseteq A$ is also uniform. The statement follows from the S-criterion. Part (b) also follows from the S-criterion.
**Axiom** **5** The atomic state *r* with Q(r)=0 and S(r)=0 is a record state, as is r+r, etc. We can take our bit state to be $I_{b}=\left\{r,r+r\right\}$. Since every information state I∈I is uniform with Q(I)=0, every information process (including $\Theta_{b}=\langle r,I_{b}\rangle$) is possible.
**Axiom** **6** Since all of the states of the form a+I are uniform, the statements in this axiom follow from the S-criterion.
**Axiom** **7** Suppose nA→nB+J. Since *J* is uniform, it must be that $nN_{A}∼nN_{B}$, from which it follows that $N_{A}∼N_{B}$. The S-criterion for $U_{A}$ and $U_{B}$ follows from a typical stability argument—that is, if nx≤ny+z for arbitrarily large values of *n*, then it must be true x≤y.
**Axiom** **8** The set M of mechanical states may be defined to include all states that can be constructed from the zero-entropy atomic state $s_{0}$ ($s_{0}+s_{0}$, $s_{0}+\left(s_{0}+s_{0}\right)$, etc.). The required properties of M follow.
**Axiom** **9** The uniform eidostate *E* has Q(E)=q≥0 and S(E)=σ≥0. Choose an integer n>σ. Now, let $e=qs_{0}$ (or e=r if q=0), $x=ns_{0}$, and $y=ns_{\lambda }$ where λ=σ/n. We find that Q(e)=q, Q(x)=Q(y)=n, S(e)=S(x)=0 and S(y)=n(σ/n)=σ. It follows that x→y and E+x↔e+y.

This model based on a simple set of atomic states has several sophisticated characteristics, including a non-trivial component of content *Q* and possible processes involving non-uniform eidostates. It is not difficult to create models of this type that are even richer and more complex. However, it may be objected that this type of model obscures one of the key features of axiomatic information thermodynamics. Here, the entropy function S does not *emerge from* the → relation among eidostates, but instead is imposed by hand to *define* → within the model. We address this deficiency in our next model.

## 13. A Simple Quantum Model

Now, we present a model for the axioms in which the entropy function does emerge from the underlying structure. The model is a simple one without mechanical states or non-trivial components of content. Every eidostate is uniform and every process is possible. On the other hand, the model is based on quantum mechanics, and so is not devoid of features of interest.

Consider an agent A that can act upon an external qubit system Q having Hilbert space Q. Based on the information the agent possesses, it may assign the states $|\psi_{1}\rangle$ or $|\psi_{2}\rangle$ to the qubit. These state vectors need not be orthogonal. That is, it may be that no measurement of Q can perfectly distinguish which of the two states is actually present. The states, however, correspond to states of knowledge of agent A, and the agent is able to perform different operations on Q depending on whether it judges the qubit to be in one state or the other. Our notion of information possessed by the agent is thus similar to Zurek’s concept of *actionable information* [22]. Roughly speaking, information is actionable if it can be used as a control variable for conditional unitary dynamics. This means that the two states of the agent’s memory ($|\mu_{1}\rangle$ and $|\mu_{2}\rangle$ in a Hilbert space A) must be distinguishable. Hence, if we include the agent in our description of the entire system, the states $|\mu_{1}\rangle ⊗|\psi_{1}\rangle$ and $|\mu_{2}\rangle ⊗|\psi_{2}\rangle$ are orthogonal, even if $|\psi_{1}\rangle$ and $|\psi_{2}\rangle$ are not.

Our model for axiomatic information thermodynamics envisions a world consisting of an agent A and an unbounded number of external qubits. Nothing essential in our model would be altered if the external systems had dimQ=d —“qudits” instead of qubits. The thermodynamic states of the qubit systems are actually states of knowledge of the agent, and so we must include the corresponding state of the agent’s memory in our physical description. The quantum state space for our model is of the form:

(63) H=A⊗Q⊗Q⊗⋯

To be a bit more rigorous, we restrict H to vectors of the form $|\Psi \rangle ⊗|0\rangle ⊗|0\rangle ⊗\cdots$, where $|\Psi \rangle \in A⊗Q^{⊗n}$ for some finite *n*, and $|0\rangle$ is a designated “zero” ket in Q. Physical states in H have 〈ΨΨ〉=1. (The space H is not quite a Hilbert space, since it is not topologically complete, but this mathematical nicety will not affect our discussion.)

Since the Qs are qubits, dimQ=2. The agent space A, however, must be infinite-dimensional, so that it contains a countably infinite set of orthogonal quantum states. These are to be identified as distinct records of the agent’s memory.

In our thermodynamic model, the elements of S (the thermodynamic “states”) are projection operators on H. For any a∈S, we have a projection on H of the form

(64) $$\Pi_{a}=|a\rangle \;\;\langle a|⊗\pi_{a}⊗|0\rangle \;\;\langle 0|⊗\cdots$$

where $|a\rangle$ is an agent state in A and $\pi_{a}$ is a non-null projection in $Q^{⊗n}$ for some finite n≥1. The value of *n* is determined by a specified integer function L(a), which we call the *length* of the state *a*. Heuristically, the thermodynamic state *a* means that the state of the world lies in the subspace $S_{a}$ onto which $\Pi_{a}$ projects. The agent’s memory is in the state $|a\rangle$ and the the quantum state of the first L(a) external qubits lies somewhere in the subspace onto which $\pi_{a}$ projects. (All of the subsequent qubits are in the state $|0\rangle$.)

Two distinct thermodynamic states correspond to orthogonal states of the agent’s memory. If a,b∈S with a≠b, then 〈ab〉=0. The projections $\Pi_{a}$ and $\Pi_{b}$ are orthogonal to each other (so that $\Pi_{a}\Pi_{b}=0$). However, it need not be the case that $\pi_{a}$ and $\pi_{b}$ are orthogonal.

Given *a*, the projection $\Pi_{a}$ projects onto the subspace $S_{a}$ The dimension of this subspace is $d_{a}=\dim S_{a}=\mathrm{Tr}\;\Pi_{a}=\mathrm{Tr}\;\pi_{a}$. Note that $d_{a}\leq 2^{L(a)}$. We will assume that there are $S_{a}$ subspaces of every finite dimension: For any integer n≥1, there exists a∈S with $d_{a}=n$.

Suppose a,b∈S correspond to $\Pi_{a}=|a\rangle \;\;\langle a|⊗\pi_{a}⊗\cdots$ and $\Pi_{b}=|b\rangle \;\;\langle b|⊗\pi_{b}⊗\cdots$. Then, we will specify that the combined state a+b corresponds to

(65) $$\Pi_{a+b}=|a+b\rangle \;\;\langle a+b|⊗\pi_{a}⊗\pi_{b}⊗\cdots \;\;.$$

Since the state a+b entails a distinct state of the agent’s knowledge, the agent state vector $|a+b\rangle$ is orthogonal to both $|a\rangle$ and $|b\rangle$. We also note that L(a+b)=L(a)+L(b).

Here is a clarifying example. Suppose a,b,c∈S. Then,

(66) $$\begin{array}{ccc} \Pi_{(a+b)+c} & = & |(a+b)+c\rangle \;\;\langle (a+b)+c|⊗\pi_{a}⊗\pi_{b}⊗\pi_{c}⊗\cdots \end{array}$$

(67) $$\begin{array}{ccc} \Pi_{a+(b+c)} & = & |a+(b+c)\rangle \;\;\langle a+(b+c)|⊗\pi_{a}⊗\pi_{b}⊗\pi_{c}⊗\cdots \end{array}$$

are distinct thermodynamic states and hence orthogonal projections in H, even though they correspond to exactly the same qubit states. The difference between (a+b)+c and a+(b+c) entirely lies in the distinct representations of the states in the agent’s memory.

The eidostates in our model are the finite nonempty collections of states in S. We can associate each eidostate with a projection operator as well. Let E={a,…} be an eidostate. We define

(68) $$\Pi_{E}=\sum_{a\in E}\Pi_{a}=\sum_{a\in E}|a\rangle \;\;\langle a|⊗\pi_{a}⊗\cdots .$$

This is a projection operator because the $\Pi_{a}$ projections are orthogonal to one another. $\Pi_{E}$ projects onto a subspace $S_{E}$, which is the linear span of the collection of subspaces $\left\{S_{a},\ldots \right\}$.

Interestingly, this subspace $S_{E}$ might contain quantum states in which the agent A is entangled with one or more external qubits. Suppose a,b∈S are associated with single-qubit projections onto distinct states $|\psi_{a}\rangle$ and $|\psi_{b}\rangle$. The eidostate E={a,b} is associated with the projection

(69) $$\Pi_{E}=\left(|a\rangle \;\;\langle a|⊗|\psi_{a}\rangle \;\;\langle \psi_{a}|+|b\rangle \;\;\langle b|⊗|\psi_{b}\rangle \;\;\langle \psi_{b}|\right)⊗\cdots ,$$

which projects onto a subspace $S_{E}$ that contains the quantum state

(70) $$|\Psi \rangle =\frac{1}{\sqrt{2}}\left(|a\rangle ⊗|\psi_{a}\rangle +|b\rangle ⊗|\psi_{b}\rangle \right)⊗\cdots .$$

In this state, the agent does not have a definite memory record state. However, if a measurement is performed on the agent (perhaps by asking it a question), then the resulting memory record *a* or *b* would certainly be found to be consistent with the state of the qubit system, $|\psi_{a}\rangle$ or $|\psi_{b}\rangle$.

Suppose we combine two eidostates A={a,…} and B={b,…}. Then, the quantum state lies in a subspace of dimension

(71) $$\begin{array}{ccc} d_{A+B}=\mathrm{Tr}\;\Pi_{A+B} & = & \mathrm{Tr}\;\sum_{a,b}\Pi_{a+b}\\ & = & \sum_{a,b}\mathrm{Tr}\;\left(|a+b\rangle \;\;\langle a+b|⊗\pi_{a}⊗\pi_{b}\right)\\ & = & \sum_{a,b}\left(\mathrm{Tr}\;\pi_{a}\right)\left(\mathrm{Tr}\;\pi_{b}\right)\\ & = & \left(\sum_{a}\mathrm{Tr}\;\pi_{a}\right)\left(\sum_{b}\mathrm{Tr}\;\pi_{b}\right)\\ & = & d_{A}\cdot d_{B}. \end{array}$$

When eidostates combine, subspace dimension is multiplicative.

It remains to define the → relation in our quantum model. We say that A→B if there exists a unitary time evolution operator U on H such that $|\Psi \rangle \in S_{A}$ implies that $U|\Psi \rangle \in S_{B}$. That is, every quantum state consistent with *A* evolves to one consistent with *B* under the time evolution U. (Note that the evolution includes a suitable updating of the agent’s own memory state.) This requirement is easily expressed as a subspace dimension criterion: A→B if and only if $d_{A}\leq d_{B}$.

We are now ready to verify our axioms.

**Axiom** **1** This follows from our construction of the eidostates E.
**Axiom** **2** All of these basic properties of the → relation follow from the subspace dimension criterion.
**Axiom** **3** If B⊊A, then $d_{A}>d_{B}$, and so A↛B.
**Axiom** **4** Again, both parts of this axiom follow from the subspace dimension criterion. If eidostate *A* is a disjoint union of eidostates $A_{1}$ and $A_{2}$, then $d_{A}=d_{A_{1}}+d_{A_{2}}$.
**Axiom** **5** Any state *r* with $d_{r}=1$ functions as a record state. We have assumed that such a state exists. We can take $I_{b}=\left\{r,r+r\right\}$. The bit process $\Theta_{b}=\langle r,I_{b}\rangle$ is natural ($r\to I_{b}$) by the subspace dimension criterion. Notice that, for any information state *I*, $d_{I}=\#\left(I\right)$.
**Axiom** **6** For any b∈S and I∈I, we have $d_{b+I}=d_{b}\cdot \#\left(I\right)$. For Part (a), we can always find a large enough information state so that $d_{b}\leq d_{a}\cdot \#\left(I\right)$. For Part (b), either $d_{a}\leq d_{b+I}$ or $d_{b+I}\leq d_{a}$.
**Axiom** **7** If ${\left(d_{A}\right)}^{n}\leq {\left(d_{B}\right)}^{n}\cdot \#\left(J\right)$ for arbitrarily large values of *n*, then $d_{A}\leq d_{B}$.
**Axiom** **8** It is consistent to take M=∅.
**Axiom** **9** All of our eidostates are uniform. For any eidostate *E*, we can choose *e* so that $d_{e}=d_{E}$. (Recall that we have assumed states with every positive subspace dimension.) If we chose x=y to be any state, then E+x↔e+y.

Our quantum mechanical model is therefore a model of the axioms of information thermodynamics. It is a relatively simple model, of course, having no non-trivial conserved components of content and no mechanical states.

In the quantum model, the entropy of any eidostate is simply the logarithm of the dimension of the corresponding subspace: $S\left(E\right)=\log d_{E}$. This is the von Neumann entropy of a uniform density operator $\rho_{E}=\frac{1}{d_{E}}\Pi_{E}$. We can, in fact, recast our entire discussion in terms of these mixed states, and this approach does yield some insights. For example, we find that the density operator $\rho_{E}$ for eidostate *E* is a mixture of the density operators for its constituent states:

(72) $$\rho_{E}=\sum_{a\in E}P\left(a|E\right)\rho_{a},$$

where P(a|E) is the entropic probability

(73) $$P\left(a|E\right)=\frac{2^{S(a)}}{2^{S(E)}}=\frac{d_{a}}{d_{E}}.$$

There are, of course, many quantum states of the agent and its qubit world that do not lie within any eidostate subspace $S_{E}$. For example, consider a state associated with a pure state projection $\Pi_{a}=|a\rangle \;\;\langle a|⊗|\psi_{a}\rangle \;\;\langle \psi_{a}|⊗\cdots$. Let $|\chi \rangle$ be a state orthogonal to $|\psi_{a}\rangle$. Then, $|a\rangle ⊗|\chi \rangle ⊗\cdots$ is a perfectly legitimate quantum state that is orthogonal to $S_{a}$ and every other eidostate subspace. This state represents a situation in which the agent’s memory record indicates that the first L(a) external qubits are in state $|\psi_{a}\rangle$, but *the agent is wrong.*

The exclusion of such physically possible but incongruous quantum states tells us something significant about our theory of axiomatic information thermodynamics. The set E does not necessarily include all possible physical situations; the arrow relations → between eidostates do not necessarily represent all possible time evolutions. Our axiomatic system is simply a theory of what transformations are possible among a collection of allowable states. In this, it is similar to ordinary classical thermodynamics, which is designed to consider processes that begin and end with states in internal thermodynamic equilibrium.

## 14. Remarks

The emergence of the entropy S, a state function that determines the irreversibility of processes, is a key benchmark for any axiomatic system of thermodynamics. Our axiomatic system does not yield a unique entropy on S, since it is based on the extension of an irreversibility function to impossible processes. However, many of our results and formulas for entropy are uniquely determined by our axioms. The entropy measure for information states, the Hartley–Shannon entropy log#(I), is unique up to the choice of logarithm base. This in turn uniquely determines the irreversibility function on possible singleton processes, since this is defined in terms of the creation and erasure of bit states. There is a unique relationship between the entropy of a uniform eidostate and the entropy of the possible states it contains. Finally, the entropic probability distribution on a uniform eidostate, which might appear at first to depend on the singleton state entropy, is nonetheless unique. It remains to be seen how the axiomatic system developed here for state transformations is related to the axiomatic system, similar in some respects, given by Knuth and Skilling for considering problems of inference [23]. There, symmetry axioms in a lattice of states give rise to probability and entropy measures. In a similar way, the “entropy first, probability after” idea presented here is reminiscent of Caticha’s “entropic inference” [24], in which the probabilistic Bayes rule is derived (along with the maximum entropy method) from a single relative entropy functional.

The entropy in information thermodynamics has two aspects: a measure of the information in a pure information state and an irreversibility measure for a singleton eidostate (the kind most closely analogous to a conventional thermodynamic state). The most general expression for the entropy of a uniform eidostate in Theorem 12 exhibits this twofold character. However, the two aspects of entropy are not really distinct in our axiomatic system. Both are based on the structure of the → relation among eidostates, which tells how one eidostate may be transformed into another by processes that may include demons.

The connection between information and thermodynamic entropy is nowhere more clearly stated than in Landauer’s principle, the minimum thermodynamic cost of information erasure. It is easy to state a theorem of our system corresponding to Landauer’s principle. Suppose a,b∈S and $I_{b}$ is a bit state. If $a+I_{b}\to b$, then it follows that S(b)≥S(a)+1. Erasing a bit state is necessarily accompanied by an increase of at least one unit in the thermodynamic entropy. More generally, suppose *A* and *B* are uniform eidostates with A→B. If we use Theorem 12 to write the entropies of the two states as

(74) $$S\left(A\right)={\langle S\rangle }_{A}+H_{A}\;\mathrm{and}\;S\left(B\right)={\langle S\rangle }_{B}+H_{B},$$

then it follows that Δ〈S〉≥−ΔH for the process taking *A* to *B*. In other words, any decrease of the “Shannon information” part of the eidostate entropy (−ΔH) must be accompanied by an increase, on average, in the thermodynamic entropy of the possible states (Δ〈S〉).

These theorems do not really constitute a “proof” of Landauer’s principle, because they depend upon the physical applicability of our axioms. On the other hand, our axiomatic framework does obviate some of the objections that have been raised to existing derivations of Landauer’s principle. Norton [25], for example, has argued that many “proofs” of the principle improperly mix together two different kinds of probability distribution, the microstate distributions associated with thermodynamic equilibrium states and the probability assignments to memory records that represent information. He calls this the problem of “illicit ensembles”. Our approach, by contrast, is not based on concepts of probability, except for those that emerge naturally from the structure of the → relation. We do not resolve thermodynamic states into their microstates at all, and we represent information by a simple enumeration of possible memory records.

The Second Law of Thermodynamics, the law of non-decrease of entropy, is the canonical example of the “arrow of time” in physics. Time asymmetry in our axiomatic theory is found in the direction of the → relation. It is an enlightening exercise to consider the axioms of information thermodynamics with the arrows reversed. Some axioms (e.g., Axioms 1 and 2) are actually unchanged by this. Others may be modified but still remain true statements in the theory. A few become false. The most striking example of the last is Axiom 3, which states that no eidostate may be transformed into a proper subset of itself. That is, no process can deterministically delete one of a list of possible states. This is the principle that leads to irreversbility in information processes, and through them, to more general irreversibility and entropy measures.

## Appendix A

This appendix provides a convenient summary of axioms of our theory.

First, we remind ourselves of a few essential definitions:

An ***eidostate*** is a set whose elements are called ***states***. The collection of eidostates is E and the collection of states is S. An element a∈S may be identified with the singleton eidostate {a}∈E.

Eidostates are combined by the Cartesian product, which we denote by the symbol +. When we combine an eidostate with itself *n* times, we use nA to denote A+(A+(A+…)). Two eidostates are ***similar*** (written A∼B) if they are made up of the same Cartesian factors, perhaps combined in a different way.

There is a relation → on E, and thus also on the singletons in S. An eidostate *A* is ***uniform*** if, for all a,b∈A, either a→b or b→a. A formal ***process*** is a pair of eidostates $\langle A,B\rangle$. We say that a process $\langle A,B\rangle$ is ***possible*** if either A→B or B→A.

A ***record state*** *r* is a state for which there exists another state *a* such that a→a+r and a+r→a (denoted a↔a+r). An ***information state*** is an eidostate containing only record states, and the set of information states is called I. A ***bit state***$I_{b}$ is an information state containing exactly two distinct record states. A ***bit process*** is a formal process $\langle r,I_{b}\rangle$, where *r* is a record state and $I_{b}$ is a bit state.

Now, we may state our axioms:

Axiom 1 (Eidostates.) E is a collection of sets called *eidostates* such that:
  (a) Every A∈E is a finite nonempty set with a finite prime Cartesian factorization.
  (b) A+B∈E if and only if A,B∈E.
  (c) Every nonempty subset of an eidostate is also an eidostate.
Axiom 2 (Processes.) Let eidostates A,B,C∈E, and s∈S.
  (a) If A∼B, then A→B.
  (b) If A→B and B→C, then A→C.
  (c) If A→B, then A+C→B+C.
  (d) If A+s→B+s, then A→B.
Axiom 3 If A,B∈E and *B* is a proper subset of *A*, then A↛B.
Axiom 4 (Conditional processes.)
  (a) Suppose $A,A^{\prime }\in E$ and b∈S. If A→b and $A^{\prime }\subseteq A$ then $A^{\prime }\to b$.
  (b) Suppose *A* and *B* are uniform eidostates that are each disjoint unions of eidostates: $A=A_{1}\cup A_{2}$ and $B=B_{1}\cup B_{2}$. If $A_{1}\to B_{1}$ and $A_{2}\to B_{2}$ then A→B.
Axiom 5 (Information.) There exist a bit state and a possible bit process.
Axiom 6 (Demons.) Suppose a,b∈S and J∈I such that a→b+J.
  (a) There exists I∈I such that b→a+I.
  (b) For any I∈I, either a→b+I or b+I→a.
Axiom 7 (Stability.) Suppose A,B∈E and J∈I. If nA→nB+J for arbitrarily large values of *n*, then A→B.
Axiom 8 (Mechanical states.) There exists a subset M⊆S of *mechanical states* such that:
  (a) If l,m∈M, then l+m∈M.
  (b) For l,m∈M, if l→m then m→l.
Axiom 9 (State equivalence.) If *E* is a uniform eidostate then there exist states e,x,y∈S such that x→y and E+x↔e+y.
